# New 4-Benzenesulfonamide Derivatives of Pyrazolo[1,5-*a*][1,3,5]triazine as Purine Bioisosteres: Development, Synthesis, and Anticancer Perspective

**DOI:** 10.2174/0109298673385989250711112625

**Published:** 2025-07-31

**Authors:** Ivan Semenyuta, Stepan Pilyo, Bohdan Demydchuk, Oleksandr Lyavinets, Volodymyr Brovarets

**Affiliations:** 1 Department of Chemistry Bioactive Nitrogen-containing Heterocyclic Bases, V.P. Kukhar Institute of Bioorganic Chemistry and Petrochemistry, Kyiv, 02094, Ukraine;; 2 Department of General Chemistry and Chemistry of Materials, Yuriy Fedkovych Chernivtsi National University, Chernivtsi, 58012, Ukraine

**Keywords:** Pyrazolo[1,5-*a*][1,3,5]triazine, anticancer agent, CDK2, cyclin-dependent kinase, cancer, purine bioisosteres, benzenesulfonamide

## Abstract

**Introduction:**

Seven new 4-[2-(dichloromethyl)pyrazolo[1,5-*a*][1,3,5]triazine derivatives were investigated for anticancer activity, possible molecular mechanisms of anticancer action, and ADMET properties.

**Methods:**

The 4-benzenesulfonamide derivatives of pyrazolo[1,5-*a*][1,3,5]triazine were synthesized using the condensation of *N*-(2,2-dichloro-1-cyanovinyl)-amides IV with 1*H*-pyrazol-5-amine. Compound antitumor activities were evaluated using the NCI-60 human cancer cell line. AutoDockTools and AutoDock Vina software were used for molecular modeling. Using the ADMETlab 3.0 and pkCSM web sources, the ADMET properties of compounds **4**, **5**, and **7** were calculated.

**Results:**

Seven new pyrazolo[1,5-*a*][1,3,5]triazine derivatives were synthesized. The compounds **4**, **5**, and **7** exhibit high activity >1 µM against leukemia, colon, and renal cancer. Compound 4 exhibited the most potent activity, with IC_50_ values of 0.32 µM against leukemia, 0.49-0.89 µM against colon cancer, and 0.92 µM against renal cancer. Molecular modeling has demonstrated a potential antitumor mechanism involving CDK. The predicted ADMET profile of compounds **4**, **5**, and **7** is favorable.

**Discussion:**

The seven novel pyrazolo[1,5-*a*][1,3,5]triazines, as purine bioisosteres, were developed, synthesized, and investigated by *in vitro* and *in silico* methods.

**Conclusion:**

Seven novel pyrazolo[1,5-*a*][1,3,5]triazine derivatives exhibited anticancer activity against the NCI-60 cancer cell lines. The compounds **4**, **5**, and **7** demonstrated strong anticancer activity, with growth inhibition (GI) values exceeding 50% across all nine cancer types tested. The most active compound, **4**, is against leukemia, colon cancer, renal cancer, and lung cancer. All compounds exhibit low toxicity, with LC_50_ values of 100 µM or greater. The molecular docking of compounds **4**, **5**, and **7** revealed the potential to inhibit cancer-associated cyclin-dependent kinases. The predicted ADMET profiles of their compounds are favorable, providing a basis for further improvement of their anticancer activity.

## 1 INTRODUCTION

**Fig. (1):**
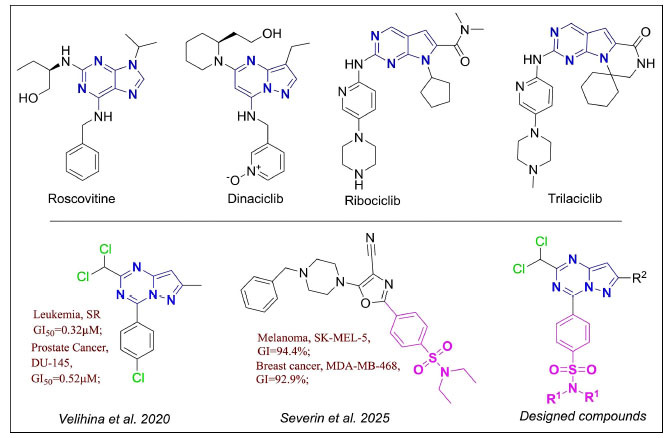
Cyclin-dependent kinase inhibitor drugs and structure-designed new compounds.

Сyclin-dependent protein kinases (CDKs) and related cyclins are essential eukaryotic cell cycle regulatory elements [1]. A living cell contains twenty CDKs, and five of them (CDK1, CDK2, CDK3, CDK4, and CDK6) are directly involved in regulating the cell cycle [2]. Cyclin-dependent kinase 2 (CDK2) regulates DNA synthesis, centriole duplication, the transition between the G1 and S phases, and the control of the G2 phase. CDK2 is located in the nucleoplasm, plasma membrane, cytoplasm, and centrosome [3]. This protein is activated and regulated by cyclins E and A, and then the active CDK-cyclin complex phosphorylates a series of cell cycle regulatory proteins. It should be noted that cyclin A is required for progression through the S phase, and cyclin E is required for transition from the G1 phase to the S phase [4]. To research data, the expression of CDK2 causes the deregulation of the cell cycle, leading to the hyperproliferation of tumor cells [5]. Besides, the deactivation of CDK2 arrests the differentiation of acute myeloid leukemia cells [6]. Bladder cancer treatment by regulating CDK2 expression can suppress tumor cell proliferation and enhance cell apoptosis [7]. CDK2 activation in cancer cells is attributed to the overexpression of cyclins A and E in several human cancers, including breast and ovarian cancer, osteosarcoma, melanoma, lung cancer, and a range of carcinomas (endometrial and thyroid) [8]. Therefore, CDK2 is a promising target for regulating cell cycle recovery in proliferating cells [9]. Recently, purine heterocycles have been the most employed scaffolds for designing CDK2 inhibitors, and these molecules show potential as anticancer agents [10]. Roscovitine is one of the earliest CDK2 inhibitors (IC_50_ = 0.1-2.7 μM) and induces apoptosis in cancer cell lines at all cell cycle phases [11]. Dinaciclib, Milciclib, Ribociclib, and Trilaciclib were synthesized with the redistribution of purine heteroatoms (similar to roscovitine), exhibiting high CDK selectivity (Fig. **1**), low toxicity, and therapeutic potential in cancer treatment [12]. So, we previously synthesized pyrazolo [1,5-*a*][1,3,^5^]triazine derivatives, which demonstrated high antitumor activity on the NCI-60 panel [13]. The most active compound, **3fa**, exhibited high activity against leukemia, colon, prostate, breast, lung, and renal cancer, with GI_50_ values ranging from 0.516 to 0.957 µM, and low-level cytotoxicity, with LC_50_ ≥ 100 µM in all cancer cell lines. In another study of our 4-(5-amino-4- cyano-1,3-oxazol-2-yl) derivatives [14] containing benzenesulfonamide groups, we found them to be highly active against all NCI-60 panels, with mean values of GI=70-104%. Therefore, in the present study, 2-(dichloromethyl)pyrazolo [1,5-*a*][1,3,^5^]triazines have been further functionalized in the 4-position by benzenesulfonamide group, and at the 7-position by methyl, phenyl, *p*-tolyl, and *p*-fluorophenyl groups (Fig. **1**) and investigated for anticancer activity (NCI- 60). Moreover, the possible molecular mechanisms of anticancer action and the ADMET profiles of these compounds were studied using molecular docking and ADMET prognosis.

## 2 MATERIALS AND METHODS

### 2.1 Chemistry

#### 2.1.1 General Information

All reagents and solvents used in the synthetic procedures were purchased from Sigma-Aldrich and used as received. The TLC method, using Silica gel 60 F254 (Merck), monitored the reaction progress. ^1^H (500 MHz) and ^13^C (100 MHz) NMR spectra of the obtained products were recorded on a Varian Unity Plus 400 spectrometer in DMSO-d_6_ or CDCl_3_ solution, with TMS as the internal standard. IR spectra were registered on a Vertex 70 FT-IR spectrometer (Bruker) using KBr pellets. Melting points were measured on a Fisher-Johns instrument. Chromatograph mass spectra were recorded on an Agilent 1100 Series high-performance liquid chromatograph equipped with a diode array detector and an Agilent LC/MS mass selective detector, allowing for fast switching between positive/negative ionization modes (chemical ionization). Elemental analyses were performed at the Analytical Laboratory of the V.P. Kukhar Institute of Bioorganic Chemistry and Petrochemistry, NAS of Ukraine. The results are consistent (±0.4%) with the calculated values.

### 2.2 Biology

#### 2.2.1 *In Vitro* Anticancer Evaluation

Under the Developmental Therapeutic Program (DTP), the National Cancer Institute (NCI) (Bethesda, Maryland, U.S.A.) investigated the anticancer activity of our synthesized compounds. The NCI Development Therapeutics Program (DTP) provides services and resources to the global academic research community to facilitate the discovery and development of new cancer therapeutic agents. An anticancer activity study was performed on the NCI-60 cell line panel (Table **S1**), which contained 59 human cancer cell lines from nine cancer types: ovarian and CNS cancer, breast and prostate cancer, leukemia, melanoma, lung, colon, and renal cancer [15]. Our synthesized compounds **1**-**7** were investigated for anticancer activity under the Developmental Therapeutic Program (DTP), The National Cancer Institute (NCI) (Bethesda, Maryland, U.S.A.) [https://dctd.cancer.gov/programs/dtp]. The NCI Developmental Therapeutics Program (DTP) provides critical services and resources to the global academic research communities and plays a central role in advancing the discovery and development of new cancer therapeutic agents by offering access to a broad range of tools, expertise, and preclinical testing services. The NCI DTP uses a panel of 60 human tumor cell lines, known as the NCI-60, to screen for potential new anti-cancer agents. NCI-60 is a panel of 60 human cancer cell lines used to test cancer drugs. It is a comprehensive cancer pharmacology database and contains lines from breast, central nervous system, colon, kidney, leukemia, lung, melanoma, ovarian, and prostate cancers. Cryopreserved batches of each cell line are stored in the NCI Developmental Therapeutics Program Tumor Repository. The anticancer screening was started by cell inoculating of each cell line into standard 96-well microtiter plates (5000-40000 cells/well) in RPMI-1640 medium (containing 5% fetal bovine serum and 2mM L-glutamine) (day 0) and preincubated for 24 h without compounds at 37°C and 5% CO_2_. Studied compounds were added (10^-5^ M) (day 1) and incubated (48 h). Next, the medium was removed, and the tumor cells were fixed *in situ*, washed, and dried (day 3). Cell density was measured using the cellular protein content determined by the sulforhodamine B assay. After incubation, the cell monolayers were fixed with 10% trichloroacetic acid and stained for 30 min. The excess dye was removed by repeatedly washing it with acetic acid. The bound stain was resolved in a 10 mM Tris-base and spectrophotometrically (λ=510 nm) measured on automated microplate readers [16].

### 2.3 Computational Modeling

#### 2.3.1 Molecular Docking Methodology

The 3D ligand structures were created and preoptimized using the ChemAxon MarvinSketch 5.5.0.1 [17] program. The Avogadro 2 (v.1.99.0) software, with the integrated force field MMFF94 [18], was employed to minimize the ligand energy. The 3D structures of proteins and ligands were prepared using the AutoDock Tools (ADT) 4.2.6 [19] program. The enzyme structures were modified by adding only polar hydrogens, and all atoms were subsequently renumbered. ADT software computed partial charges (Gasteiger method) for the ligands and kinase structures. The refined 3D structures of enzymes and ligands were saved as pdbqt files. For docking investigations, we used the AutoDock Vina 1.2.5 program [20], which operates with a grid map of 35 × 35 × 35 Å and a grid spacing of 0.375 Å. Discovery Studio 2019 software [21] was used to visualize enzyme-ligand interactions.

#### 2.3.2 Evaluating ADMET

The ADMETlab 3.0 Web server [22] was employed to predict the ADMET properties of compounds **4**, **5**, and **7** compared to Roscovitine. This ADMET predicted module comprises a series of high-quality models trained using the DMPNN framework, enabling computations and predictions for twenty-one physicochemical properties, nineteen medicinal chemistry properties, 34 ADME types, 36 toxicity species, and eight toxicophore rules. Additionally, the pkCSM web server was utilized to predict several ADMET properties [23].

## 3 RESULTS AND DISCUSSION

### 3.1 Chemistry

**Scheme 1:**
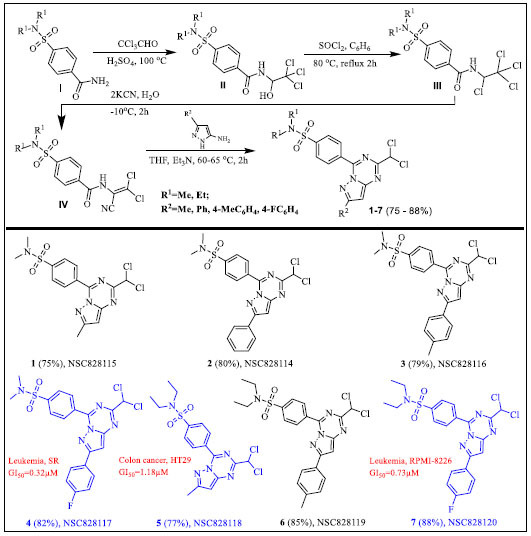
Synthesis and structures of new pyrazolo[1,5-*a*][1,3,5]triazines **1-7**.

Scheme **1** demonstrates the general synthesis of pyrazolo [1,5-*a*][1,3,^5^]triazines **1 - 7** by condensation of the relevant 4-dialkyl-sulfamoylbenzamides. The condensation of commercially available 4-dialkyl-sulfamoylbenzamides **I** with chloral was directed to the formation of *N*-(2,2,2-trichloro-1-hydroxyethyl) amides **II**, which were then reacted with thionyl chloride to produce *N*-(1,2,2,2-tetrachloroethyl) amides **III**. These compounds were used directly in the next step without purification. The subsequent substitution of the chlorine atom in amides **III** with a cyano group led to the synthesis of *N*-(2,2-dichloro-1-cyanovinyl)-amides **IV**. The final step involved heterocyclization, which was achieved through the condensation of 1*H*-pyrazol-5-amine with *N*-(2,2-dichloro-1-cyanoethenyl)-4-(dialkylsulfamoyl)benzamide **IV** (Scheme **1**). The key new compounds **1-7**, with a dichloromethyl group in position 2, were obtained in good yields (75-88%) (Table **S2**) [13, 24, 25].

#### 3.1.1 General Procedure of Synthesis for Pyrazolo [1,5-*a*] [1,3,^5^]triazines 1-7

To a solution of amide **IV** (0.01 mol) in THF (10 mL), pyrazolamines (0.01 mol), followed by triethylamine (0.01 mol), were added. The mixture was stirred at room temperature for 24 hours and then heated to 55-60°C for 2 hours. After evaporating the solvent under vacuum, the residue was triturated with water to yield a crude product, which was then separated and recrystallized for analysis.

*4-[2-(Dichloromethyl)-7-methylpyrazolo [1,5-*a*][*1, 3,5*]triazin-4-yl]-N,N-dimethylbenzenesulfonamide* **1.** Yield 75%, mp 208-209°C. IR (KBr) υ cm^-1^: 3007, 1601, 1497, 1480, 1338 (SO_2_), 1163 (SO_2_), 713, 595. ^1^H NMR (500 MHz, DMSO-*d_6_*) δ_H_: 2.26 - 2.32 (6H, m), 2.34 (3H, s), 6.67 (1H, s), 7.24 (1H, s), 7.86 (2H, d, *J*=8.6 Hz), 8.67 (2H, d, *J*=8.5 Hz). ^13^C NMR (126 MHz, DMSO) δ: 14.6, 37.5, 39.5, 70.8, 97.8, 127.4, 131.9, 133.5, 149.8, 152.3, 152.9, 157.2, 159.0. LC/MS, m/z: 400.0 [M]^+^.

*4-[2-(Dichloromethyl)-7-phenylpyrazolo [1,5-*a*][*1, 3,5*]triazin-4-yl]-N,N-dimethylbenzenesulfonamide **2**.* Yield 80%, mp 241-242°C. IR (KBr) υ cm^-1^: 3001, 1599, 1482, 1458, 1439, 1345 (SO_2_), 1165 (SO_2_), 944, 768, 747, 703, 689, 662, 605, 584, 530. ^1^H NMR (500 MHz, DMSO-*d_6_*) δ_H_: 2.79 (6H, s), 7.37 (1H, s), 7.50 (1H, s), 7.50 - 7.70 (3H, m), 8.10 (2H, d, *J*=8.3 Hz), 8.16 (2H, d, *J*=7.6 Hz), 9.05 (2H, d, *J*=7.0 Hz). ^13^C NMR (126 MHz, DMSO-*d_6_*) δ: 37.8, 39.5, 40.0, 71.1, 95.3, 127.2, 127.8, 129.3, 130.5, 131.3, 132.3, 133.7, 139.2, 150.9, 153.4, 157.7, 158.9. LC/MS, m/z: 462.0 [M]^+^.

*4-[2-(Dichloromethyl)-7-(4-methylphenyl)pyrazolo [1,5-*a*][*1,3,5*]triazin-4-yl]-N,N-dimethylbenzenesulfonamide **3**.* Yield 79%, mp 243-244°C. IR (KBr) υ cm^-1^: 2965, 1601, 1481, 1450, 1342 (SO_2_), 1165 (SO_2_), 785, 753, 697, 659, 594. ^1^H NMR (500 MHz, DMSO-*d_6_*) δ_H_: 2.37 (3H, s), 2.73 (6H, s), 7.33 (2H, d, *J*=7.9 Hz), 7.48 (1H, s), 7.52 (1H, s), 8.04 (2H, d, *J*=7.9 Hz), 8.10 (2H, d, *J*=8.5 Hz), 9.02 (2H, d, *J*=8.5 Hz). ^13^C NMR (101 MHz, DMSO-*d_6_*) δ: 21.5, 38.1, 40.6, 71.4, 95.3, 127.3, 128.0, 128.8, 130.1, 132.6, 134.0, 139.3, 140.6, 151.1, 153.5, 157.9, 159.2. LC/MS, m/z: 478.0 [M+2H]^+^.

*4-[2-(Dichloromethyl)-7-(4-fluorophenyl)pyrazolo [1,5-*a*][*1,3,5*]triazin-4-yl]-N,N-dimethylbenzenesulfonamide **4**.* Yield 82%, mp 198-199°C. IR (KBr) υ cm^-1^: 2983, 1608, 1585, 1494, 1483, 1451, 1345 (SO_2_), 1239, 1165 (SO_2_), 1158, 850, 788, 773, 752, 696, 662, 595. ^1^H NMR (500 MHz, DMSO-*d_6_*) δ_H_: 2.73 (6H, s), 7.40 (2H, t, *J*=9.9, 7.8 Hz), 7.49 (1H, s), 7.60 (1H, s), 8.10 (2H, d, *J*=8.6 Hz), 8.25 (2H, d, *J*=8.6, 5.6 Hz), 9.01 (2H, d, *J*=8.5 Hz). ^13^C NMR (126 MHz, DMSO) δ: 37.5, 39.5, 70.8, 95.0, 116.0, 116.1, 117.7, 127.5, 127.7, 129.3, 132.1, 133.4, 138.9, 150.7, 153.2, 157.5, 157.7. ^19^F NMR (376 MHz, DMSO-*d_6_*) δ: -111.0. LC/MS, m/z: 480.0 [M]^+^.

*4-[2-(Dichloromethyl)-7-methylpyrazolo [1,5-*a*][*1, 3,5*] triazin-4-yl]-N,N-diethylbenzenesulfonamide **5**.* Yield 77%, mp 156-157°C. IR (KBr) υ cm^-1^: 2987, 1602, 1503, 1484, 1467, 1448, 1331 (SO_2_), 1205, 1157 (SO_2_), 1016, 774, 746, 704, 682, 667, 591. ^1^H NMR (500 MHz, DMSO-*d_6_*) δ_H_: 1.08 (7H, t, *J*=7.1 Hz), 2.54 (3H, s), 3.25 (4H, q, *J*=7.1 Hz), 6.86 (1H, s), 7.43 (1H, s), 8.10 (2H, d, *J*=8.3 Hz), 8.84 (2H, d, *J*=8.4 Hz). ^13^C NMR (101 MHz, DMSO-*d_6_*) δ: 14.1, 14.6, 39.5, 41.9, 70.7, 97.7, 126.5, 131.8, 133.0, 143.5, 149.7, 152.7, 157.1, 158.8. LC/MS, m/z: 428.0 [M]^+^.

*4-[2-(Dichloromethyl)-7-(4-methylphenyl)pyrazolo [1,5-*a*][*1,3,5*]triazin-4-yl]-N,N-diethylbenzenesulfonamide **6**.* Yield 85%, mp 233-234°C. IR (KBr) υ cm^-1^: 2985, 1601, 1492, 1480, 1448, 1335 (SO_2_), 1159 (SO_2_), 785, 743, 691, 591. ^1^H NMR (500 MHz, CDCl_3_) δ_H_: 1.19 (6H, t, *J*=7.1 Hz), 2.44 (3H, s), 3.33 (4H, q, *J*=7.1 Hz), 6.77 (1H, s), 7.08 (1H, s), 7.32 (2H, d, *J*=7.8 Hz), 7.93 (2H, d, *J*=8.3 Hz), 8.07 (2H, d, *J*=8.8 Hz), 9.16 (2H, d, *J*=8.7 Hz). ^13^C NMR (126 MHz, CDCl_3_) δ: 14.4, 21.6, 42.4, 70.4, 77.2, 95.1, 127.0, 127.2, 128.6, 129.9, 132.5, 133.2, 140.9, 145.0, 151.3, 153.2, 158.2, 160.2. . LC/MS, m/z: 504.0 [M]^-^.

*4-[2-(Dichloromethyl)-7-(4-fluorophenyl)pyrazolo [1,5-*a*][*1,3,5*]triazin-4-yl]-N,N-diethylbenzenesulfonamide **7**.* Yield 88%, mp 204-205°C. IR (KBr) υ cm^-1^: 2993, 1606, 1586, 1497, 1482, 1450, 1335 (SO_2_), 1236, 1157 (SO_2_), 846, 787, 745, 692, 590. ^1^H NMR (500 MHz, DMSO-*d_6_*) δ_H_: 1.10 (6H, t, *J*=7.1 Hz), 3.27 (4H, q, *J*=7.1 Hz), 7.38 (2H, t, *J*=8.9 Hz), 7.48 (1H, s), 7.57 (1H, s), 8.14 (2H, d, *J*=8.4 Hz), 8.22 (2H, d, *J*=8.7, 5.6 Hz), 8.97 (2H, d, *J*=8.5 Hz). ^13^C NMR (101 MHz, DMSO-*d*_6_) δ: 14.2, 39.5, 42.0, 70.8, 95.0, 116.0, 116.2, 126.8, 127.7, 129.2, 129.3, 132.2, 133.0, 143.7, 150.7, 153.2, 157.5, 157.6, 162.1. ^19^F NMR (376 MHz, DMSO-*d*_6_) δ: -111.0. LC/MS, m/z: 508.0 [M]^-^.

The structure of the synthesized compounds **1-7** is consistent with the IR, ^1^H, ^13^C, and ^19^F NMR spectroscopy data and LC-MS spectrometry. All results of spectral studies are presented in the Supporting Information (Figs. **S1-S30**). The ^1^H NMR spectra of compounds **1-7** show signals corresponding to all protons of the aromatic and aliphatic substituents. Notably, the protons of aromatic substituents bound to the triazine ring are downfield-shifted by approximately 1.0 ppm compared to those of unsubstituted aromatic substituents, which typically appear in the 7.3-9.1 ppm range. The signals of the protons in the CHCl_2_ group and the pyrazole cycle appear as singlets at 6.6-7.5 ppm and 7.1-7.6 ppm, respectively. The signals of the carbon nuclei of the pyrazole ring and the CHCl_2_ group in the ^13^C NMR spectra are 94.9-97.8 ppm and 70.4-71.3 ppm, respectively. For fluorine-containing compounds **4** and **7**, the signals of fluorine nuclei in the ^19^F NMR spectra appear at -111.00 ppm. In the IR spectra of compounds **1-7**, intense absorption bands of the SO_2_ group are observed at 1157-1165 cm^-1^ and 1331-1345 cm^-1^.

### 3.2 Biology

#### 3.2.1 Anticancer Screening of Compounds 1-7 (One-dose Assay)

At the first stage, the results of anticancer activity (10^-5^ M, one-dose assay) were obtained for all pyrazolo [1,5-*a*][1,3,^5^]triazines, statistically processed, and presented in Table **1**. The results demonstrate that all compounds have antitumor activity against NCI-60 cell lines. According to the mean values of total growth inhibition, the studied compounds can be ranked in order of activity: **4 > 5 > 7 > 1 > 2 > 3 > 6**. Complete anticancer data for the one-dose assay of all compounds are shown in Table **S3**.

Compounds **4, 5,** and **7** displayed higher inhibitory activity than other derivatives against cell lines of all subpanels. So, compound **4** showed high activity against colon cancer (mean GI=97%), breast cancer (GI=94%), ovarian cancer (92%), and leukemia (90%). Compound **5** is the most active against colon cancer (95%), breast cancer (83%), and leukemia (85%). Compound **7** exhibits high activity against breast cancer (GI = 89%), colon cancer (GI = 85%), and prostate cancer (GI = 83%). Additionally, according to the results of a one-dose assay (10^-5^ M), the most active compounds are **4**, **5**, and **7**; their average inhibitory activity is 3-7 times higher than that of other compounds. Next, compounds **4, 5,** and **7** were screened using the NCI-60 five-dose assay.

#### 3.2.2 Anticancer Screening of Compounds 4, 5, 7 (Five-dose Assay)

Table **2** presents the results of statistical analyses on anticancer compounds **4, 5,** and **7**. These results demonstrate micromolar mean values of GI_50_ (for nine types of cancer) for compounds **4**, **5**, and **7**, at 2.13 µM, 2.94 µM, and 3.39 µM, respectively, representing a series of activities: **4 > 5 > 7**. The mean values of TGI for these compounds are -21.19 µM, 60.18 µM, and 54.92 µM. It should be noted that compounds **4, 5,** and **7** exhibit low cytotoxicity for all subpanels; the mean LC_50_ values are ≥100 µM, except compound **4** (lung cancer - 40.44 µM; colon cancer - 39.15 µM).

Thus, all compounds exhibited high anticancer activity, with compound **4** being the most active. The GI_50_ values for the subpanels most sensitive to it are 1.16 µM for colon cancer, 1.73 µM for leukemia, 1.81 µM for ovarian cancer, and 1.84 µM for lung cancer. Further, results of growth inhibition of most sensitive cancer cells for compounds **4**, **5**, and **7** shown in Fig. (**2**) demonstrate high inhibition activity (less than 1 µM) compound **4** on leukemia cells - K-562 (GI_50_=0.93 µM), MOLT-4 (GI_50_=0.89 µM), SR (GI_50_=0.32 µM); colon cancer cells - HCT-116 (GI_50_=0.99 µM), HT29 (GI_50_=0.49 µM) and SW-620 (GI_50_=0.89 µM) and renal cancer cells ACHN with GI_50_=0.93 µM. Important features of their anticancer activity are the anti-leukemia selectivity of compounds **4** (0.32 µM) and **7** (0.73 µM), as well as the low cytotoxicity of compounds **4**, **5**, and **7**, with LC_50_ values ≥ 100 µM.

Thus, compounds **4, 5**, and **7** inhibited all cancer cell lines, suggesting a most general molecular mechanism of anticancer activity. The investigation yields positive results for the inhibition of CDK2 by pyrazolo [1,5-*a*][1,3,^5^]triazine derivatives, with an IC_50_ of approximately 2 μM [26]. The CDK inhibitors are effective targets for treating cancer because disorders in cyclins, CDKs, and CDK regulators are common in most cancers. Therefore, the potential mechanism of the anticancer activity of compounds **4**, **5**, and **7** was investigated using molecular docking into a series of CDKs.

### 3.3 Computational Modeling

#### 3.3.1 Molecular Docking Study

Molecular docking for compounds was performed using the following structures of human Cyclin-dependent kinases (CDKs): CDK1 (PDB ID: 6GU6), CDK2 (PDB ID: 3DDQ), CDK4 (PDB ID: 7SJ3), and CDK6 (PDB ID: 8I0M). The 3D CDK structures were obtained from the Global Protein Data Bank (RCSB) [27]. Table **3** shows the interaction features of compounds in the active centers of CDK1, CDK2, CDK4, and CDK6.

Compounds Binding Energy (∆G), kcal/molCDK1CDK2CDK4CDK64- 8.6- 8.8- 10.1- 9.05- 8.1- 8.2- 8.5- 8.17- 8.4- 8.7- 8.6- 8.8Dinaciclib- 9.1-Roscovitine- 8.8-Abemaciclib- 10.9-NJ6 ^a^- 9.1^a^ 2-[(4-aminocyclohexyl)amino]-7-cyclopentyl-*N,N*-dimethyl- pyrrolo [2,3-*d*]pyrimidine-6-carboxamide (PDB ID: 8I0M).

The verification of molecular docking was performed by redocking co-crystalized ligands: dinaciclib, roscovitine, abemaciclib, and 2-[(4-aminocyclohexyl)amino]-7-cyclopentyl-*N,N*-dimethyl-pyrrolo [2,3-*d*]pyrimidine-6-carboxamide into the ATP-binding sites of human CDK1, CDK2, CDK4, and CDK6, respectively. The calculated binding energy (∆G) ranged from -8.8 to -10.9 kcal/mol, and the RMSD values were 1.367-1.815 Å. The docking results (Table **3**) indicated that compounds **4**, **5**, and **7** formed high-energy ligand-receptor complexes within the ATP-binding centers of CDKs, with estimated binding energies ranging from -8.1 to -10.1 kcal/mol. Compounds **4** and **7** exhibit the highest activity (∆G = -8.8, -8.7 kcal/mol) with CDK2, comparable to the co-crystalized roscovitine (∆G = -8.8 kcal/mol). Also, compound **4** forms ligand-kinase complex with CDK6 (∆G= -9.0 kcal/mol) similar to co-crystalized 2-[(4-aminocyclohexyl)-amino]-7-cyclopentyl-*N*, *N*-dime-thyl-pyrrolo [2,3-*d*]pyrimidine-6-carboxamide (∆G= -9.1 kcal/mol). Complexation compounds **5** and **7** with CDKs happen with the lower energy: CDK1 (-8.1 and -8.4 kcal/mol), CDK2 (-8.2 and -8.7 kcal/mol), CDK4 (-8.5 and -8.6 kcal/mol) and CDK6 (-8.1 and -8.8 kcal/mol). Therefore, compounds **4** and **7** formed the most energetically favorable complexes in the ATP-binding sites of CDK2 and CDK6, with binding free energies (∆G) of -8.8, -8.7, and -10.1 kcal/mol, respectively. It should be noted that compound **4's** high binding energy of complexation (compared to roscovitine) is correlated to its high *in vitro* anticancer activity. Furthermore, Fig. (**3**) illustrates the interaction of compound **4** with the ATP-binding site of CDK2.

Three hydrogen bonds stabilize the formed complex: between the oxygen atoms of the sulfonamide group and the amino acid residues LYS33 (1.87Ǻ) and ASP145 (2.20Ǻ), between the nitrogen of the pyrazolo [1,5-*a*][1,3,^5^]triazine ring and the amino acid GLY13 (2.67Ǻ). Additionally, in complex stabilization, the four electrostatic interactions are between the fluorine atom and amino acid HIS84 (2.82 Å); among the sulfur atom of the sulfonamide group and PHE80 (3.91 Å); and between the phenyl ring and amino acid ASP86 (4.29 Å). Besides, in complex stabilization, it participates in a series of hydrophobic interactions with amino acid residues ILE10, VAL18, LYS129, and ALA 144 (3.66 - 5.40Ǻ). Therefore, the molecular docking of compound **4** demonstrated a possible mechanism of its anticancer action, involving binding to the ATP-binding site of CDK2, similar to roscovitine and ATP (*e.g.*, PDB ID: 8FP5) (Fig. **4**).

#### 3.3.2 Structure-activity Relationship

**Fig. (3):**
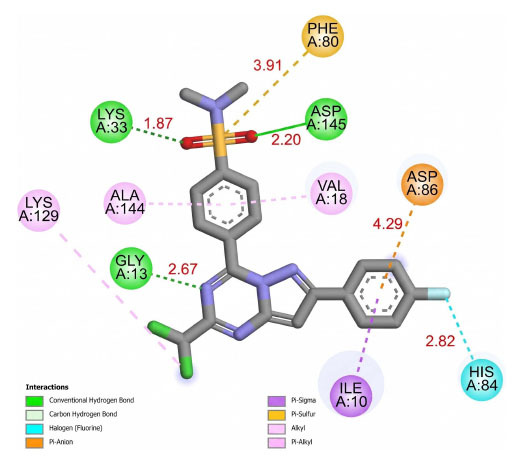
Molecular docking of the compound **4** in the CDK2 ATP-binding site.

**Fig. (4):**
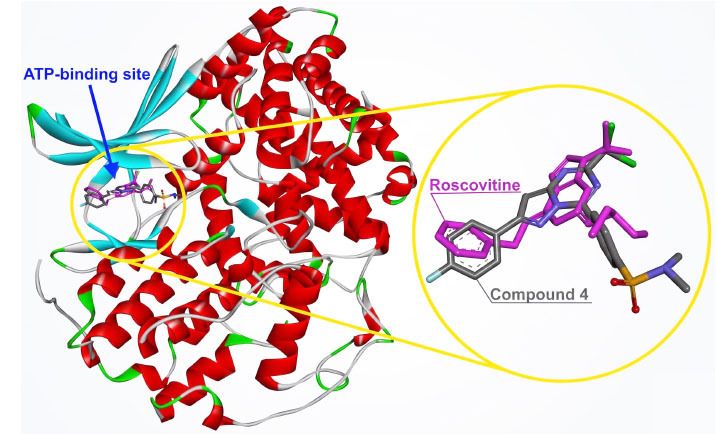
The docking position of сompound **4** (grey) in the ATP-binding site of CDK2 along with Roscovitine (violet) (PDB ID: 3DDQ).

**Fig. (5):**
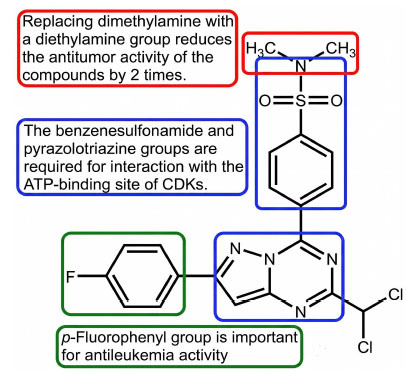
Structure-activity relationship (SAR) analysis from the compound 4.

The structure-activity relationship (SAR) analysis (Fig. **5**) of compounds **4**, **5**, and **7** reveals the high activity of p-fluorophenyl-containing compounds **4** and **7** against leukemia cells - SR (0.32 µM) and RPMI-8226 (0.73 µM), respectively. The high activity of fluorine- containing compounds **4** and **7** is in good agreement with the anticancer activity of known fluorine-containing anticancer drugs - 5-fluorouracil, Abemaciclib, Lorlatanib, Entrectinib, Dacomitinib, Sunitinib, *etc.* Thus, according to the results of molecular docking (Fig. **3**), the benzenesulfonamide group and pyrazolo [1,5-*a*][1,3,^5^]triazine moiety are directly involved in binding to the ATP-binding site of CDKs. This fact is confirmed by studying the complexation of roscovitine-CDK2 and dinaciclib-CDK1 in the crystal structures PDB ID: 3DDQ and PDB ID: 6GU6.

Replacing the dimethylamine group with diethylamine reduces mean activity by 1.6 times (with 3.4 µM to 2.1 µM) of compound **7** compared to **4**. In this case, antileukemia activity was significantly reduced by 8 times in cell line SR and by 4 times in cell line MOLT-4. For all compounds, modifying the dimethylsulfonamide groups on the diethylsulfonamide reduces average anticancer activity by 20%. Depending on substituents at position 7 of pyrazole - methyl, phenyl, *p*-tolyl, and *p*-fluorophenyl allow the following series of anticancer activity compounds (dimethyl-benzenesulfonamides) to be constructed: **4** > **1** > **2** > **3**. The anticancer activity of diethylbenzenesulfonamides decreases in the series **5** ~ **7** > **6**. Next, compared to roscovitine, ADMET studies were conducted on compounds **4**, **5**, and **7**.

#### 3.3.3 ADMET Evaluation

Table **4** presents the ADMET profiles of compounds **4**, **5**, **7**, and roscovitine (for comparison) as predicted by the ADMETlab 3.0 web server. The compounds exhibited tolerated molecular properties, including a count of rotatable bonds (5-7), and a count of hydrogen bond donors (0) and acceptors (7). The surface area of synthesized compounds **4**, **5**, and **7** is 167-200 Å^2^, slightly above the roscovitine value. The LogP coefficient lipophilicity of compounds is accepted as being related to membrane permeability, and the series can be given as follows: compound **5** (3.609) > compound **4** (3.810) > roscovitine (3.996) > compound **7** (4.895). The BBB and CNS penetrability of the studied compounds is below the permissible threshold (logBBB permeability < -1, logCNS permeability < -3) and below the corresponding roscovitine values. The absorption results indicate that the compounds are well-permeabilized through intestinal cell membranes (logCaco-2 permeability > 5.15) and that they do not interact with P-glycoprotein as a biological barrier. The excretion compounds were predicted to have a total clearance for compound **4** at an average level (>5 ml/min/kg) and below the average level for compounds **5** and **7** (4.3-4.6 ml/min/kg). Additionally, the results for the compounds' half-lives were obtained, which range from 0.674 to 1.092 (for roscovitine, 0.81), representing an average value. The compounds' toxicology properties are represented by positive hepatotoxicity (similar to roscovitine) and a high maximum recommended tolerated dose (0.301-0.775); this dose for roscovitine is 0.832. The values of the oral acute toxic doses of compounds **4**, **5**, and **7** are comparable to those of roscovitine. Also shown in Fig. (**6**) is the ADMET profile of compound **4** calculated by the ADMETlab 3.0 web server.

## 4 DISCUSSION

Therefore, CDKs play a significant role in regulating the cell cycle and proliferation, and are excellent therapeutic targets for anticancer treatment [28]. However, most first-generation CDK inhibitors have not been approved for treatment due to their significant toxicity and lack of selectivity [29]. Also, these low-selective CDK inhibitors cause medication resistance [30]. These obstacles are being overcome by the development of more selective CDK inhibitors. Currently, the U.S. Food and Drug Administration has approved three specific CDK4/6 inhibitors—Palbociclib, Ribociclib, and Abemaciclib—for the first-line treatment of HR+/HER2- breast cancer [31]. Also, the development of combination therapy strategies has decreased the toxicity level and side effects of pan-CDK inhibitors. Thus, pan-CDK inhibitors are gaining high potential in cancer treatment using a combination therapy approach [32]. Therefore, developing new derivatives with pyrazolo [1,5-*a*][1,3,^5^]triazine moiety as potential inhibitors of CDKs is relevant.

## CONCLUSION

Seven novel pyrazolo [1,5-*a*][1,3,5]triazine derivatives, as purine bioisosters, were developed and synthesized, and anticancer activity was investigated against the NCI-60 cancer cell lines. The final 7-substituted 4-[2-(dichloromethyl)-pyrazolo [1,5-*a*][1,3,^5^]triazin-4-yl]-*N,N*-dialkylbenzenesulfonamides 1-7 were synthesized to a four-stage procedure: condensation of 4- dialkylsulfanylbenzamides **I** with chloral, reaction the *N*-(2,2,2-trichloro-1-hydroxyethyl) amides **II** with thionyl chloride, the subsequent substitution of the chlorine atom in *N*-(1,2,2,2-tetrachloroethyl) amides **III** with a cyano group led to the synthesis of *N*-(2,2-dichloro-1-cyanoethenyl)-4-(dialkylsulfanyl)benzamides **IV** and their following heterocyclization by the condensation of with relevant 1*H*-pyrazol-5-amines. The antitumor activity of compounds 1-7 was estimated against nine types of cancer (NCI-60 cell lines). The synthesized compounds **4, 5,** and **7** demonstrated high anticancer activity with GI values greater than 50% against all nine types of cancer. The most active compound **4**, shows high GI_50_ values against leukemia cells (K-562, 0.93 µM; MOLT-4, 0.89 µM; SR, 0.32 µM); colon cancer cells (HCT-116, 0.98 µM; HT29, 0.49 µM; SW-620, 0.89 µM); and renal cancer cells (ACHN, GI_50_ = 0.92 µM). Notably, the compounds display low toxicity with LC_50_ values ≥ 100 µM, and compounds **4** and **7** exhibit selectivity for anti-leukemia activity. The SAR analysis of compounds **1-7** showed the importance of the p-fluorophenyl group in the display of anticancer activity for compounds **4** and **7**. The molecular docking of compounds **4**, **5**, and **7** revealed the potential to inhibit cancer-associated cyclin-dependent kinases, including CDK1, CDK2, CDK4, and CDK6. The predicted ADMET profile compounds **4**, **5**, and **7** are favorable. Thus, compound **4** exhibited the highest *in vitro* anticancer activity (NCI-60) and represents the basis for further improvement of pyrazolo [1,5-*a*][1,3,^5^]triazine derivatives as purine bioisosteres.

## STUDY LIMITATIONS

**Chemical Aspects:** First, in our study, among compounds **1-7**, only dimethylamine and diethylamine derivatives were synthesized and studied. Secondly, the active compounds **4** and **7** are *p*-fluorophenyl derivatives. However, it would be interesting to study compounds, such as *p*-chlorophenyl derivatives, as well as those with halogens in the ortho and meta positions.

**Biological Aspects:** An *in vitro* anticancer activity study was performed on the NCI-60 cell line panel, containing 60 human cancer cell lines. However, no studies have been conducted on the compounds on normal human cells (non-cancerous) to obtain data on the cytotoxicity of compounds. Also, to confirm/refute the docking results, we believe it is possible to conduct *in vitro* enzymatic studies of active compounds **4**, **5**, and **7** using the human cyclin-dependent kinase CDK2.

## AUTHORS’ CONTRIBUTIONS

The authors confirm their contribution to the paper as follows: study conception and design: IS and VB; data collection: IS, SP; Data Analysis or Interpretation: IS, VB; methodology: IS, SP, BD; investigation: IS, SP, BD, OL; draft manuscript: IS, VB, SP, BD. All authors reviewed the results and approved the final version of the manuscript.

## Figures and Tables

**Fig. (2):**
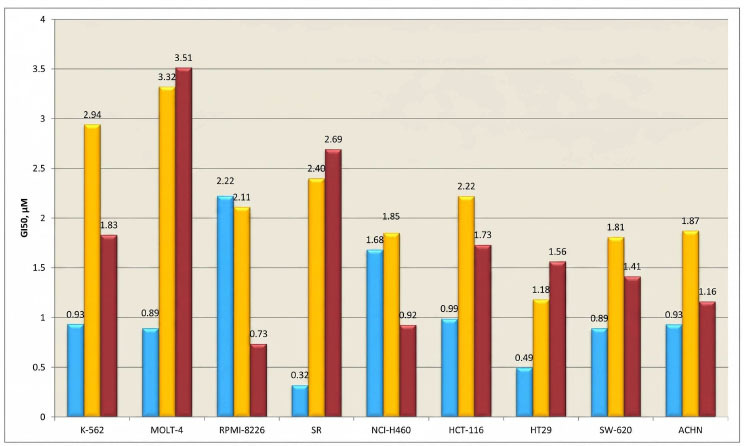
The most sensitive cancer cell lines and GI_50_ values for compounds **4** (blue), **5** (yellow), and **7** (red).

**Fig. (6):**
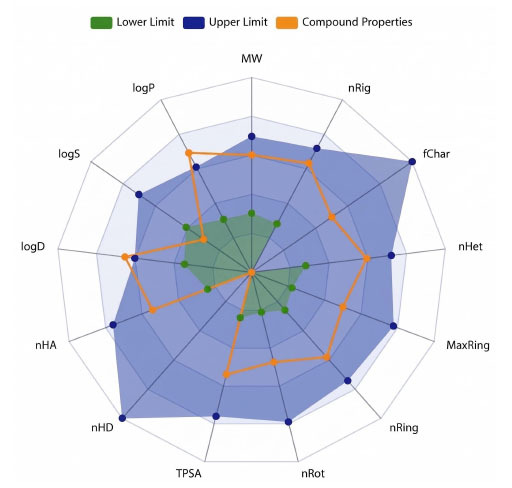
ADMET profile of compound **4** (by ADMETlab 3.0).

**Table 1:** Mean values of growth inhibition of nine NCI-60 subpanels for compounds 1-7, GI, %.

**NCI-60 subpanels**	**Compounds / GI, %**						
	**1**	**2**	**3**	**4**	**5**	**6**	**7**
Total	47.24±5.17	75.51±3.49	87.50±2.83	13.08±3.27	26.62±5.15	96.90±2.05	29.83±4.69
Leukemia	25.22±9.08	64.50±8.13	69.39±6.86	9.38±2.84	14.42±10.48	83.88±4.66	34.50±12.23
NSCLC	50.93±6.34	85.66±3.12	96.21±5.20	12.55±6.57	25.88±9.87	103.29±2.39	31.84±10.37
Colon Cancer	24.53±5.83	65.60±8.02	80.52±5.30	2.72±6.26	5.24±5.16	91.85±7.55	15.19±3.92
CNS Cancer	59.40±5.06	81.47±3.71	91.02±4.63	24.57±6.71	42.66±7.71	97.31±1.90	35.67±7.07
Melanoma	56.37±10.07	59.08±15.34	91.15±6.59	17.86±8.50	38.64±11.71	99.70±4.90	53.08±7.77
Ovarian Cancer	48.09±8.62	80.33±5.81	92.57±6.21	7.81±3.49	21.97±4.73	100.85±3.72	37.75±6.20
Renal Cancer	65.10±9.09	83.54±7.30	88.02±6.54	26.28±11.11	50.41±11.82	97.79±3.36	32.52±13.88
Prostate Cancer	56.27±15.81	83.46±5.97	88.47±4.50	16.34±14.67	23.50±10.72	99.94±1.15	17.29±1.83
Breast Cancer	39.24±16.52	75.92±11.81	90.11±6.09	5.67±15.92	17.17±18.00	97.49±6.58	10.65±13.54

**Table 2:** Mean values GI_50_, TGI, and LC_50_ of nine NCI-60 subpanels for compounds **4**, **5**, and **7**.

**NCI-60 subpanels**	**Compounds/Anticancer Activity, µM**								
	**4**			**5**			**7**		
	**GI_50_**	**TGI**	**LC_50_**	**GI_50_**	**TGI**	**LC_50_**	**GI_50_**	**TGI**	**LC_50_**
Total	2.13 ±0.27	21.19 ±10.72	≥100	2.94 ±0.43	60.18 ±14.62	≥100	3.39 ±0.43	54.92 ±9.13	≥100
Leukemia	1.73 ±0.40	>100	>100	2.67 ±0.24	≈100	>100	3.06 ±1.49	68.41 ±21.89	>100
NSCLC	1.84 ±0.15	7.41 ±2.47	40.44 ±12.63	2.65 ±0.47	26.55 ±14.73	≥100	2.49 ±0.32	39.59 ±16.11	≥100
Colon Cancer	1.16 ±0.17	3.68 ±0.44	39.41±17.18	1.76 ±0.13	4.33 ±0.67	≥100	1.69 ±0.11	46.36 ±20.50	≥100
CNS Cancer	2.53 ±0.66	15.93 ±8.97	82.96 ±16.43	3.42 ±0.79	≥100	>100	4.55 ±1.65	56.01 ±21.56	>100
Melanoma	1.96 ±0.28	6.50 ±1.73	≥100	2.72 ±0.66	≥100	>100	4.33 ±0.79	88.85 ±11.92	>100
Ovarian Cancer	1.81 ±0.20	6.39 ±0.75	≥100	2.33 ±0.20	52.16 ±6.65	>100	3.08 ±0.43	86.12 ±14.99	≥100
Renal Cancer	3.85 ±1.30	21.07 ±12.42	≥100	2.26 ±0.24	53.14 ±18.94	>100	4.09 ±1.84	30.34 ±16.30	≥100
Prostate Cancer	1.92 ±0.47	7.42 ±1.62	≥100	5.95 ±2.06	5.95 ±2.06	>100	1.98 ±0.90	10.79 ±11.05	≥100
Breast Cancer	2.41 ±0.33	22.32 ±17.04	≥100	2.71 ±0.42	≥100	≥100	5.24 ±12.92	67.82 ±21.15	>100

**Table 3:** The molecular docking of the ligands into the ATP-binding sites of CDKs and the redocking results.

**Compounds**	**Binding energy (∆G), kcal/mol**			
	**CDK1**	**CDK2**	**CDK4**	**CDK6**
**4**	- 8.6	**- 8.8**	- 10.1	**- 9.0**
**5**	- 8.1	- 8.2	- 8.5	- 8.1
**7**	- 8.4	**- 8.7**	- 8.6	- 8.8
Dinaciclib	- 9.1	-	-	-
Roscovitine	-	**- 8.8**	-	-
Abemaciclib	-	-	- 10.9	-
NJ6 ^a^	-	-	-	**- 9.1**

**Note:** ^a^ 2-[(4-aminocyclohexyl)amino]-7-cyclopentyl-N, N-dimethylpyrrolo[2,3-*d*]pyrimidine-6-carboxamide (PDB ID: 8I0M).

**Table 4:** ADMET characteristics of compounds **4**, **5**, and **7** in comparison with roscovitine.

**Parameter**	**Compounds**			
	**4**	**5**	**7**	**Roscovitine**
**Physicochemical Properties**				
Molecular weight, g/mol	479.04	427.06	507.07	354.22
Surface area, A^2 a^	187.68	167.55	200.41	153.06
Rotatable bond count	5	6	7	8
Hydrogen bond acceptor count	7	7	7	7
Hydrogen bond donor count	0	0	0	3
logP	3.810	3.609	4.895	3.996
Water solubility, log mol/L	-5.711	-4.900	-6.209	-4.849
**Absorption**				
Caco-2 permeability, log cm/s	-4.689	-4.863	-4.759	-5.291
Inhibitor of P-glycoprotein	No	No	No	Yes
Substrate of P-glycoprotein	No	No	No	No
**Distribution**				
BBB permeability ^a^	-1.704	-1.481	-1.752	-1.199
CNS permeability ^a^	-3.518	-3.121	-3.495	-2.846
**Metabolism**				
CYP2d_6_ substrate	No	No	No	No
CYP3A4 substrate	Yes	Yes	Yes	No
CYP1A2 inhibitor	No	Yes	No	Yes
CYP2C19 inhibitor	No	No	Yes	No
CYP2C9 inhibitor	Yes	Yes	Yes	Yes
CYP2d_6_ inhibitor	No	Yes	Yes	Yes
CYP3A4 inhibitor	Yes	No	No	Yes
**Excretion**				
Plasma clearance, ml/min/kg	5.496	4.601	4.315	8.298
Half-life of the drug, hour	0.674	1.092	0.885	0.81
**Toxicity**				
Rat Oral Acute Toxicity (LD50), mol/kg ^a^	2.189	2.231	2.196	2.249
Human Hepatotoxicity ^a^	Yes	Yes	Yes	Yes
Max. tolerated dose (human), log mg/kg/day ^a^	0.759	0.301	0.775	0.832

**Note:** ^a^prediction by the PKCSM web server.

## Data Availability

All data obtained or analyzed during this study are included in this published article and the SI.

## LIST OF ABBREVIATIONS

CDK: Сyclin-dependent Protein Kinase
NCI: National Cancer Institute (Bethesda, Maryland, U.S.A.)
MMFF94: Merck Molecular Force Field
NCI-60: Cell Line Panel Containing 60 Human Cancer Cell Lines from Nine Cancer Types
RMSD: Root Mean Square Deviation
ADT: AutoDock tools
