# Supplementary material for: New 4-Benzenesulfonamide Derivatives of Pyrazolo[1,5-a][1,3,5]triazine as Purine Bioisosteres: Development, Synthesis, and Anticancer Perspective
Source: Curr Med Chem. 2025 Jul 31;33(12):2406–20. doi: 10.2174/0109298673385989250711112625 (PMC13478943; doi:10.2174/0109298673385989250711112625)
Supplement: Supplementary file 1 [file CMC-33-12-2406_SD1.pdf]

Supplementary Material

New 4-Benzenesulfonamide Derivatives of Pyrazolo[1,5-a] [1, 3, 5]tri-azine as Purine Bioisosteres: Development, Synthesis, and Anticancer Perspective

Ivan Semenyuta[1,*], Stepan Pilyo[1], Bohdan Demydchuk[1], Oleksandr Lyavinets[2] and Volodymyr Brovarets[1]

[1]Department of Chemistry Bioactive Nitrogen-containing Heterocyclic Bases, V. P. KukharInstitute of Bioorganic Chemistry and Petrochemistry, Kyiv, 02094, Ukraine; [2]Department of General Chemistry and Chemistry of Materials, Yuriy Fedkovych Chernivtsi National University, Chernivtsi, 58012, Ukraine

Table S1. The specification NCI-60 Cancer Cell Line Panel including the 59 cell lines for academic and non-profit institutions.

	Type	Cell Line	Sex	Age	Histologic Type	Comments	Treatment	Source
1	COLON	COLO 205	M	70	Adenocarcinoma	Can Res 38: 1345-1455, 1978		
2	COLON	HCC-2998			Carcinoma		N	
3	COLON	HCT-15			Adenocarcinoma	Can Res 39: 1020-1025, 1970		
4	COLON	HCT-116			Carcinoma	Can Res 41: 1761-1766, 1981		
5	COLON	HT-29	F	44	Adenocarcinoma, GR III	Human Tumor Cells *In Vitro* : 115- 159, 1975		Primary
6	COLON	SW-620	M	51	Adenocarcinoma	Can Res 36: 4562-4569, 1976		Metastasis
7	CNS	SF-268	F	24	Anaplastic Astrocytoma	Acta Neuropathol 75: 92-103, 1987		
8	CNS	SF-295	F	67	Glioblastoma- Multiforme	Acta Neuropathol 75: 92-103, 1987		
9	CNS	SF-539				J Neuropathol Exp Neu-rol 40: 201- 229, 1981		
10	CNS	SNB-19	M	47	Glioblastoma (Same as U251)	Cancer 47: 255, 1981	N	
11	CNS	SNB-75	F		Astrocytoma		N	
12	CNS	U-251	M	75	Glioblastoma (Same as SNB-19)	J Neuropathol Exp Neu-rol 40: 410- 427, 1981		
13	LEUKEMIA	CCRF-CEM	M	4	Acute Lymphoblastic Leukemia	Can Res 18: 522-529, 1965		
14	LEUKEMIA	HL-60(TB)	F	36	Promyelocytic Leukemia	Nature 270: 347-349, 1977		PBL

15	LEUKEMIA	K-562	F	53	Chronic Myelogenous Leukemia	Blood 45: 321-334, 1975		Pleural Effusion
16	LEUKEMIA	MOLT-4	M	19	Acute Lymphoblastic Leukemia	JCNI 49:891-895, 1972		PB
17	LEUKEMIA	RPMI-8226	M	61	Myeloma	Proc Soc Exp Biol Med 125: 1246- 1250, 1967		PB
18	LEUKEMIA	SR	M	11	Large Cell, Immunoblastic		Y	
19	LUNG	A549 ATCC	M	58	Adenocarcinoma	JNCI 51: 1417-1423, 1973		Primary
20	LUNG	EKVX	M		Adenocarcinoma			
21	LUNG	HOP-62	F	60	Adenocarcinoma		N	
22	LUNG	HOP-92	M	62	Large Cell, Undifferentiated		N	
23	LUNG	NCI-H23			Adenocarcinoma	Can Res 45: 2913-2923, 1985	N	
24	LUNG	NCI-H226			Squamous	Can Res 45: 2913-2923, 1985		
25	LUNG	NCI-H322M			Small Cell Bronchioalveolar Carcinoma		N	
26	LUNG	NCI-H460	M		Large Cell Carcinoma	Science 246: 491-494, 1989	N	Pleural Effusion
27	LUNG	NCI-H522			Adenocarcinoma	Can Res 45: 2913-2923, 1985		
28	MAMMARY	MCF-7	F	69	Adenocarcinoma	JCNI 51:1409-1417, 1973	Y	
29	MAMMARY	HS 578T	F	74	Carcinosarcoma	JNCI 58:1795-1806, 1977		Primary
30	MAMMARY	MDA-MB-231	F	51	Adenocarcinoma	JNCI 53: 661-674, 1974, Licensed to MDA	Y	
31	MAMMARY	MDA-MB- 468	F	51	Adenocarcinoma	Cancer Res 40: 3118-3129, 1980, Licensed to MDA		
32	MAMMARY	BT-549	F	72	Papillary Infiltrating Ductal Carcinoma	No Publication		Metastasis
33	MAMMARY	T-47D	F	54	Infiltrating Ductal Carcinoma	Eur J Cancer 15: 659-670, 1979		Not for commercial use
34	MELANOMA	LOX IMVI			Malignant Amelanotic Melanoma	Int J Cancer 41:442-449, 1988		
35	MELANOMA	M14						
36	MELANOMA	MALME-3M	M	43	Malignant Melanoma	Human Tumor Cells *In Vitro* , 115- 159, 1975		Metastasis
37	MELANOMA	MDA-MB-435	F	31	Adenocarcinoma	Can Res 40: 3118-3129, 1980, Licensed to MDA	N	

38	MELANOMA	SK-MEL-2	M	60	Malignant Melanoma	Human Tumor Cells *In Vitro* , 115- 159, 1975		Metastasis
39	MELANOMA	SK-MEL-5			Malignant Melanoma	PNAS 73: 3278-3282, 1976		Metastasis
40	MELANOMA	SK-MEL-28			Malignant Melanoma	PNAS 73: 3278-3271, 1976		
41	MELANOMA	UACC-62						
42	MELANOMA	UACC-257						
43	OVARIAN	IGR-OV1	F	47	Cystoadenocarcinoma	Can Res 45: 4970-4979, 1985	N	
44	OVARIAN	NCI/ADR- RES	F		Adenocarcinoma	JNCI 90(11): 6/3/1998		See Note *
45	OVARIAN	OVCAR-3	F	60	Adenocarcinoma	Can Res 43: 5379-5389, 1983	Y	Ascites
46	OVARIAN	OVCAR-4	F	42	Adenocarcinoma	Sem Oncol 11: 285-298, 1984	Y	
47	OVARIAN	OVCAR-5	F	67	Adenocarcinoma	Sem Oncol 11: 285-298, 1984	N	
48	OVARIAN	OVCAR-8	F	64	Adenocarcinoma	Sem Oncol 11: 285-298, 1984	Y	
49	OVARIAN	SK-OV-3	F	64	Adenocarcinoma	Human Tumor Cells *In Vitro* , 115- 159, 1975	Y	Ascites
50	PROSTATE	DU-145	M	69	Carcinoma	Int J Cancer 21: 274-281, 1978	Y	
51	PROSTATE	PC-3	M	62	Adenocarcinoma	Invest Urol 17: 16-23, 1979	Y	Metastasis
52	RENAL	786-O	M	58	Adenocarcinoma	In Vitro 12: 623-627, 1976	N	
53	RENAL	A498	F	52	Adenocarcinoma	JNCI 51: 1417-1423, 1973		
54	RENAL	ACHN	M	22	Renal Cell Carcinoma	Can Res 42: 4948-4953, 1973		
55	RENAL	CAKI-1	M	49	Clear Cell Carcinoma	Human Tumor Cells *In Vitro* , 115- 159, 1975	Y	Metastasis
56	RENAL	RXF 393	M	54	Poorly Differentiated Hypernephroma	Contrib Oncol 42, 1992, Licensed to CRL	N	
57	RENAL	SN12C	M	43	Carcinoma	Can Res 46: 4109-4115, 1986		
58	RENAL	TK-10	M	43	Spindle Cell Carcinoma	Can Res 47: 3856-3862, 1987	N	
59	RENAL	UO-31			Carcinoma		N	

Table S2. Nomenclature and chemical structures of synthesized compounds 1-7.

No	NSC number	Structure	Name
**1**	828115	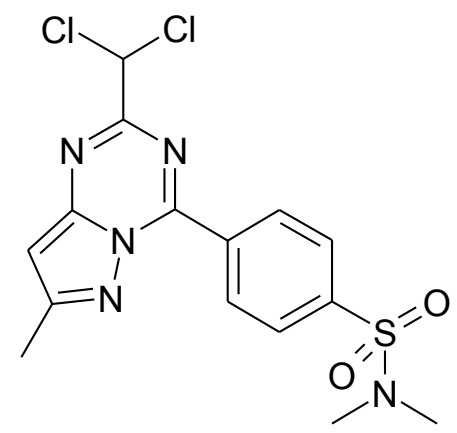	4-(2-Dichloromethyl-7-methyl-pyrazolo[1,5-a][1,3,5]triazin-4-yl)-N,N-dimethyl-benzenesulfonamide
**2**	828114	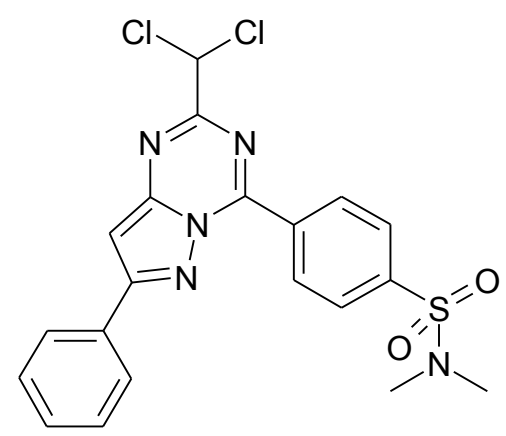	4-(2-Dichloromethyl-7-phenyl-pyrazolo[1,5-a][1,3,5]triazin-4-yl)-N,N-dimethyl-benzenesulfonamide
**3**	828116	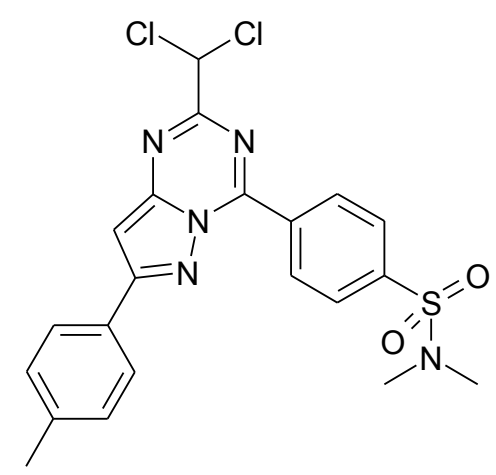	4-(2-Dichloromethyl-7-p-tolyl-pyrazolo[1,5-a][1,3,5]triazin-4-yl)-N,N-dimethyl-benzenesulfonamide
**4**	828117	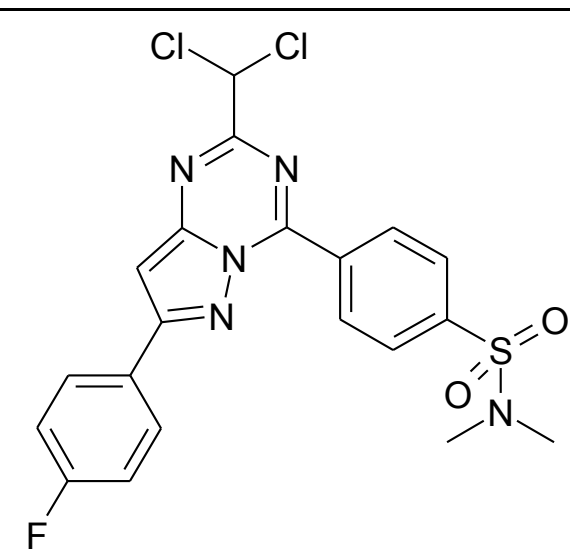	4-[2-Dichloromethyl-7-(4-fluoro-phenyl)-pyrazolo[1,5-a][1,3,5]triazin-4-yl]-N,N-dimethyl-benzenesulfonamide
**5**	828118	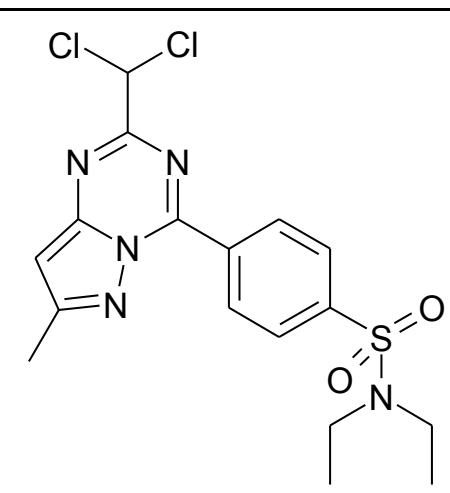	4-(2-Dichloromethyl-7-methyl-pyrazolo[1,5-a][1,3,5]triazin-4-yl)-N,N-diethyl-benzenesulfonamide
**6**	828119	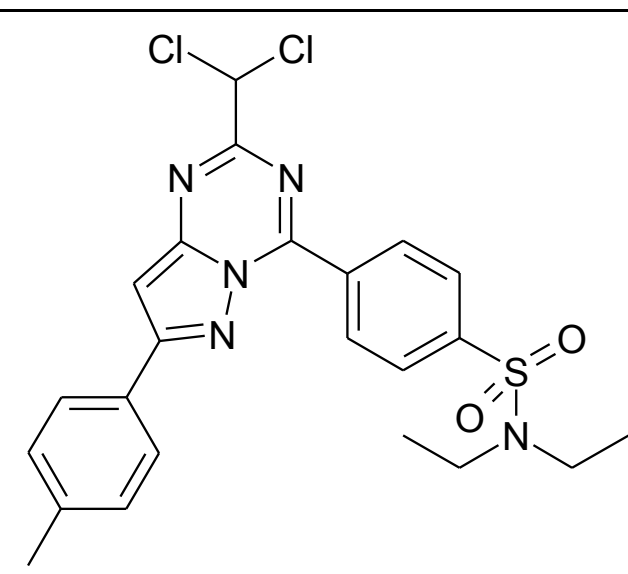	4-(2-Dichloromethyl-7-p-tolyl-pyrazolo[1,5-a][1,3,5]triazin-4-yl)-N,N-diethyl-benzenesulfonamide

**7**	828120	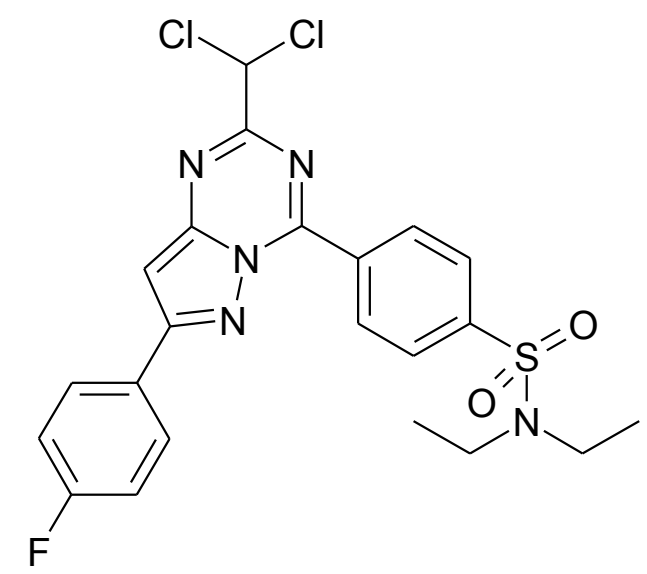	4-[2-Dichloromethyl-7-(4-fluoro-phenyl)-pyrazolo[1,5-a][1,3,5]triazin-4-yl]-N,N-diethyl-benzenesulfonamide

IR, ^{1}H, ^{13}C, ^{19}F NMR and LC/MS Spectra of compounds 1-7

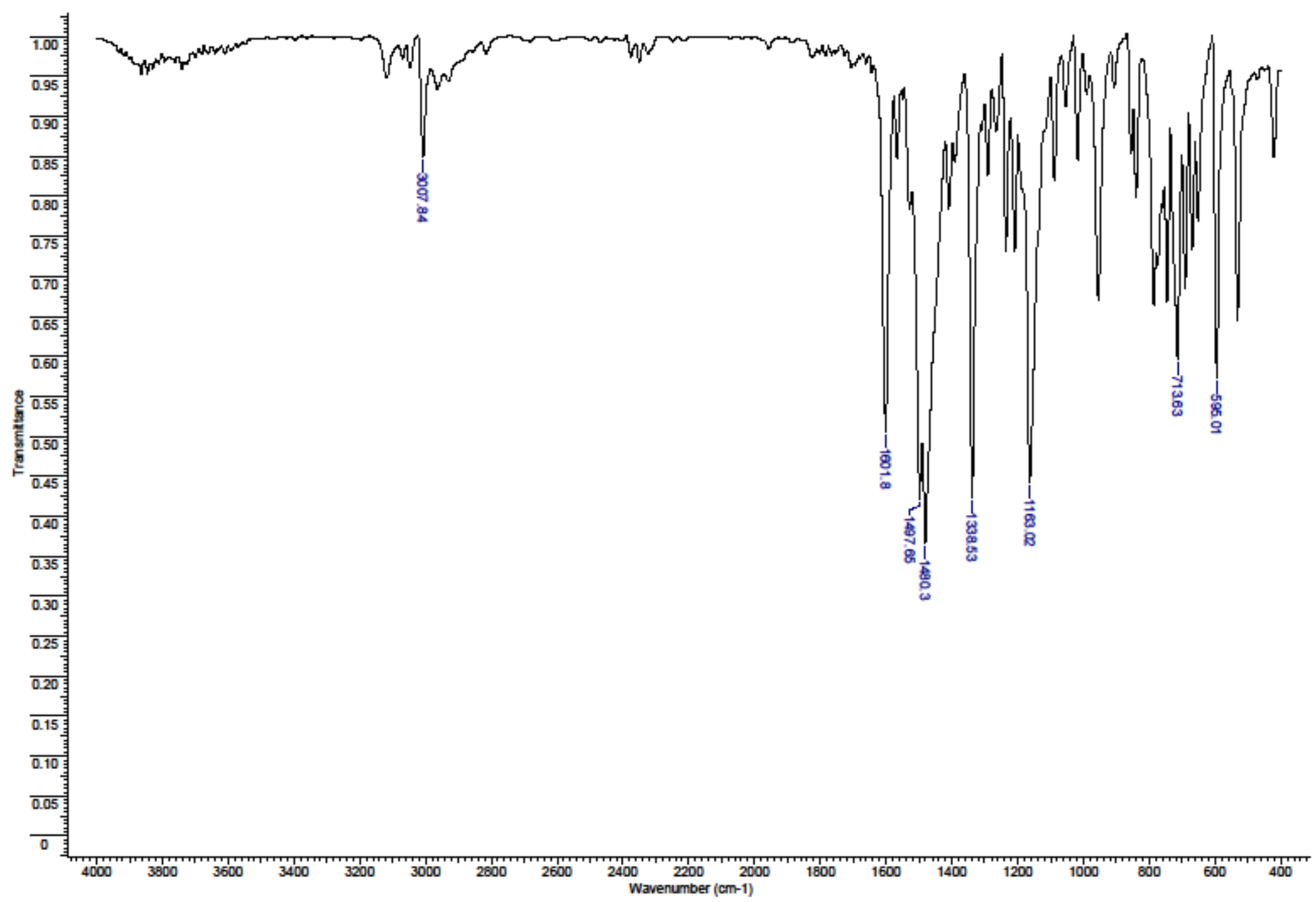

Fig. S1. IR spectrum of compound **1**.

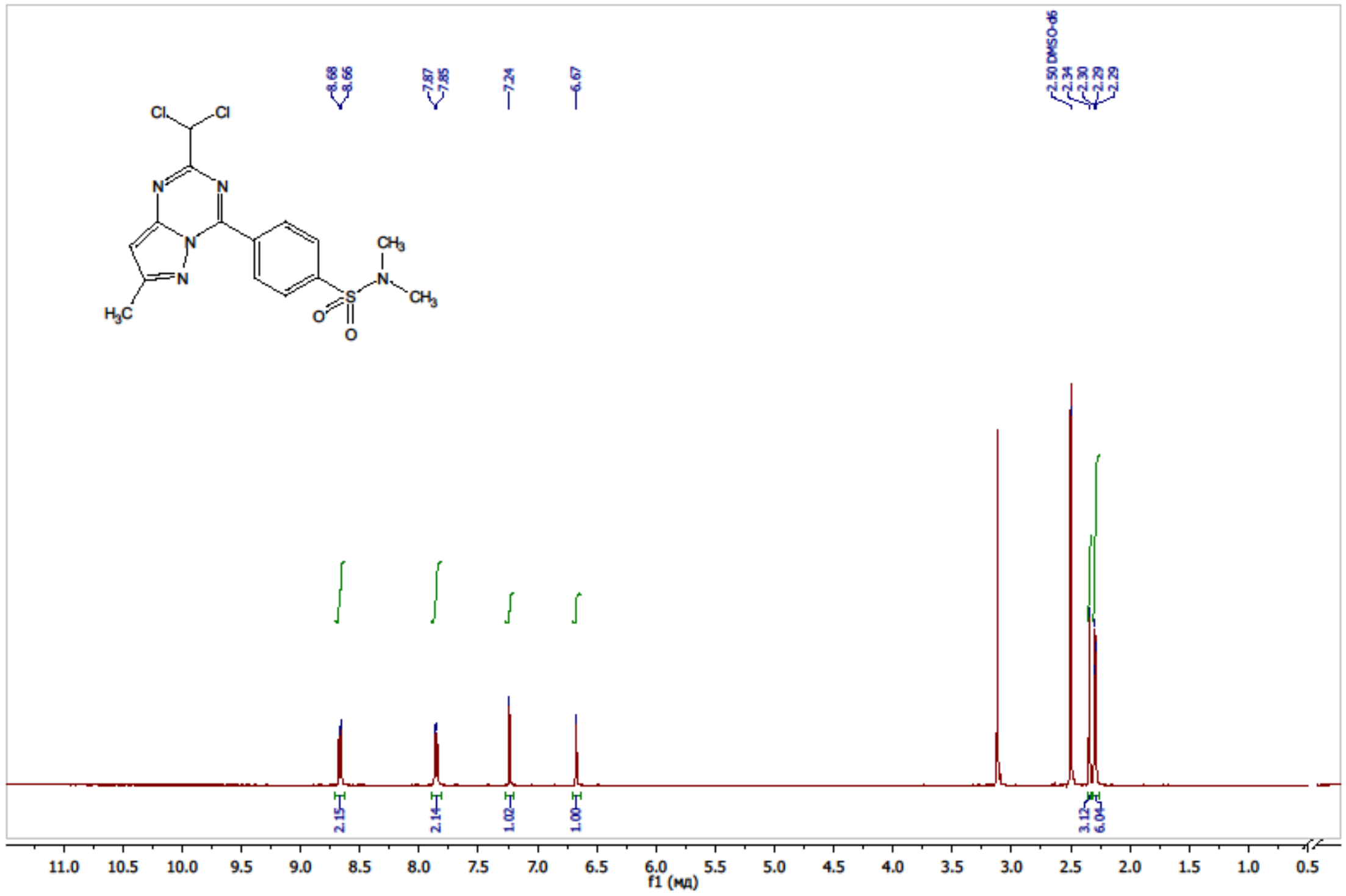

Fig. S2. ^{1}H NMR spectrum of compound **1**.

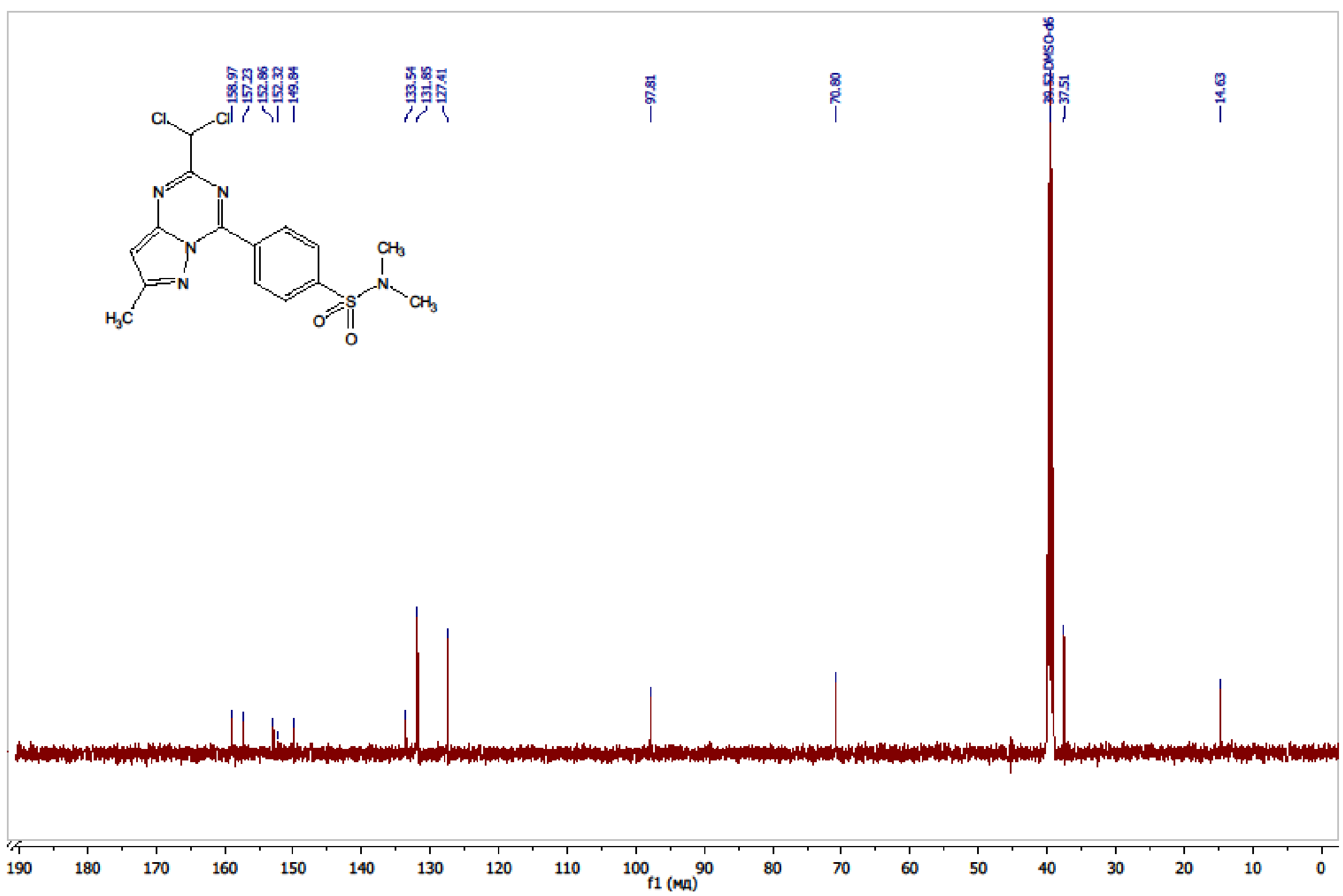

Fig. S3. ^{13}C NMR spectrum of compound **1**.

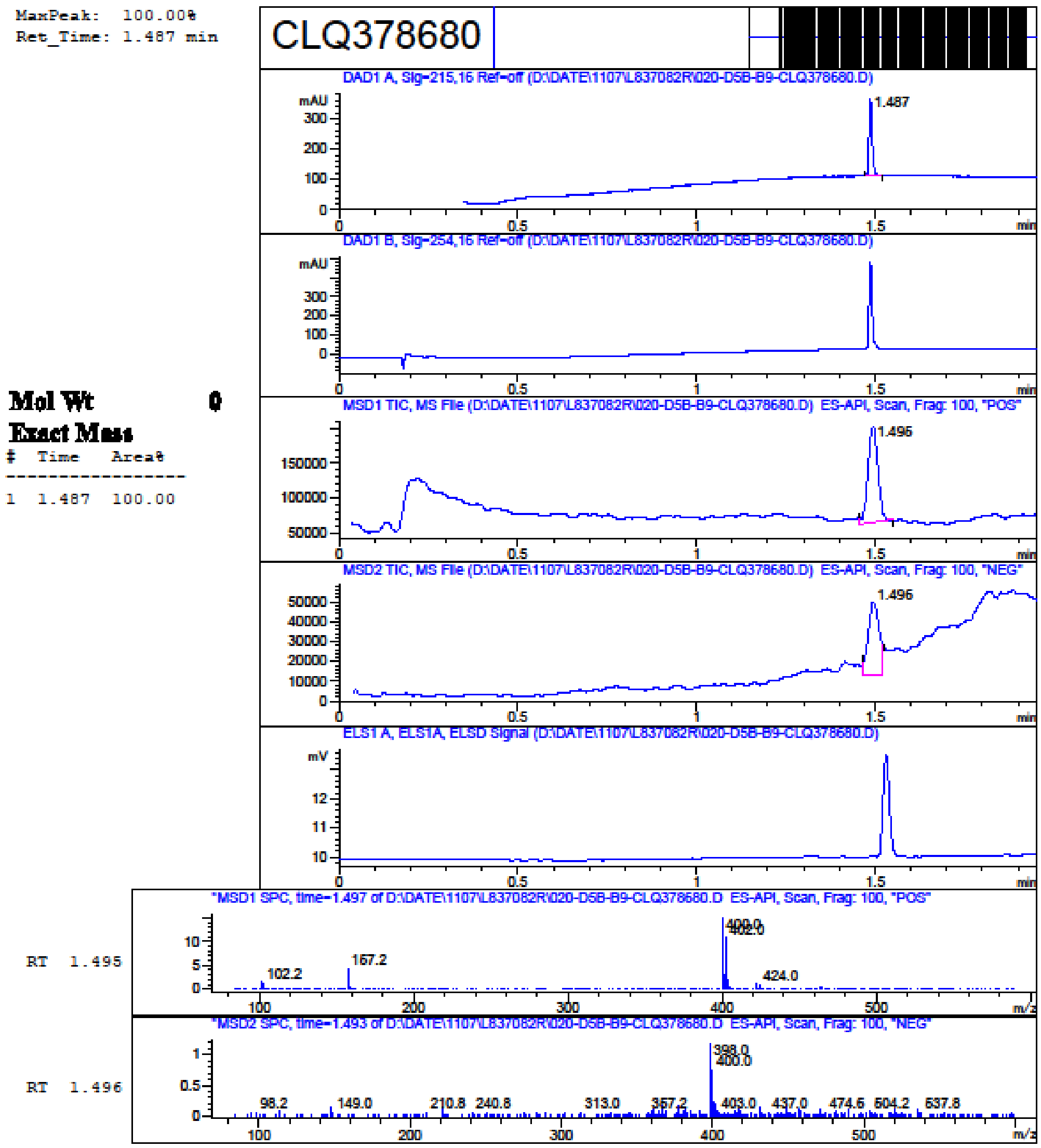

Fig. S4. LC/MS spectrum of compound **1**.

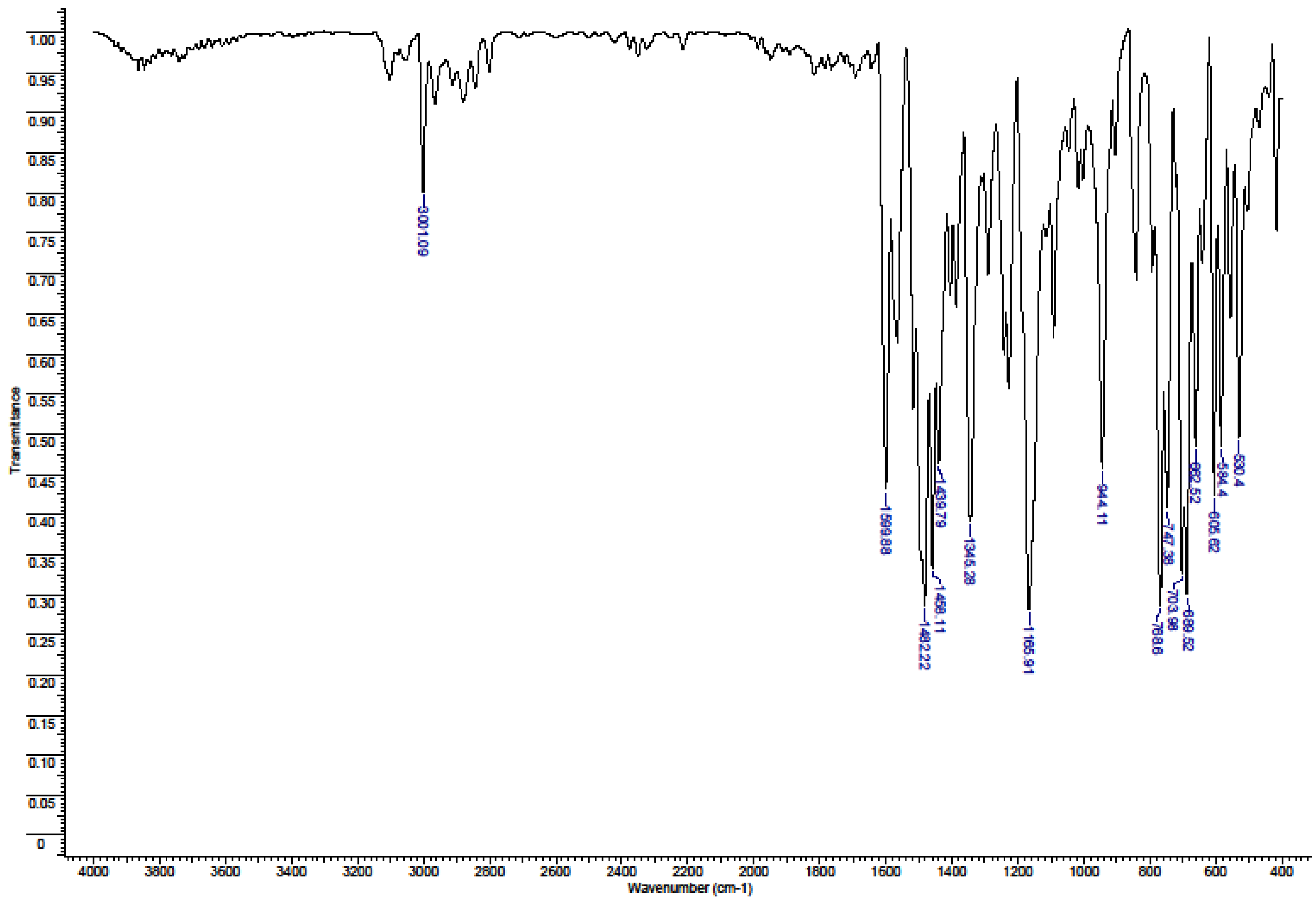

Fig. S5. IR spectrum of compound **2**.

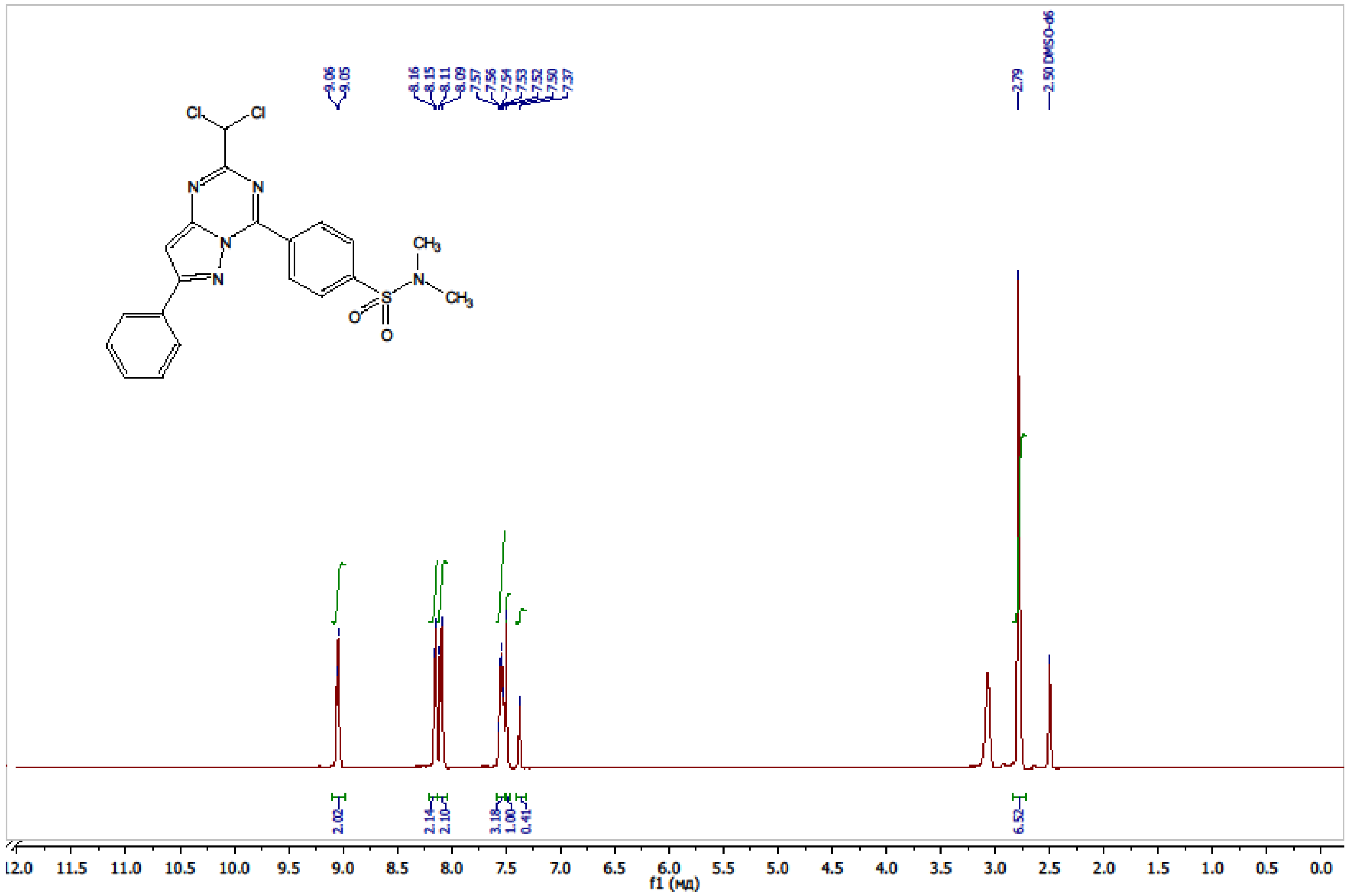

Fig. S6. ^{1}H NMR spectrum of compound **2**.

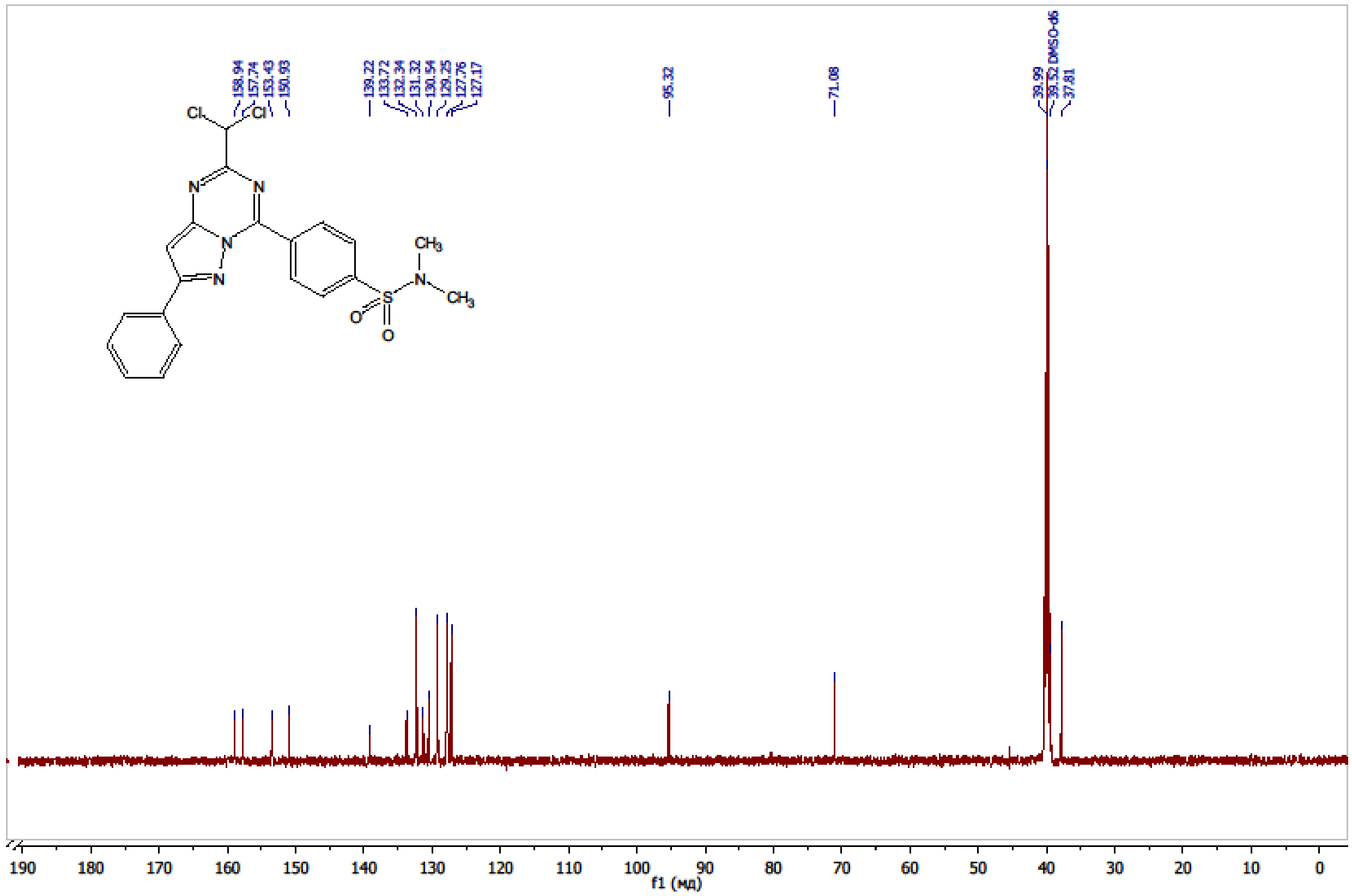

Fig. S7. ^{13}C NMR spectrum of compound **2**.

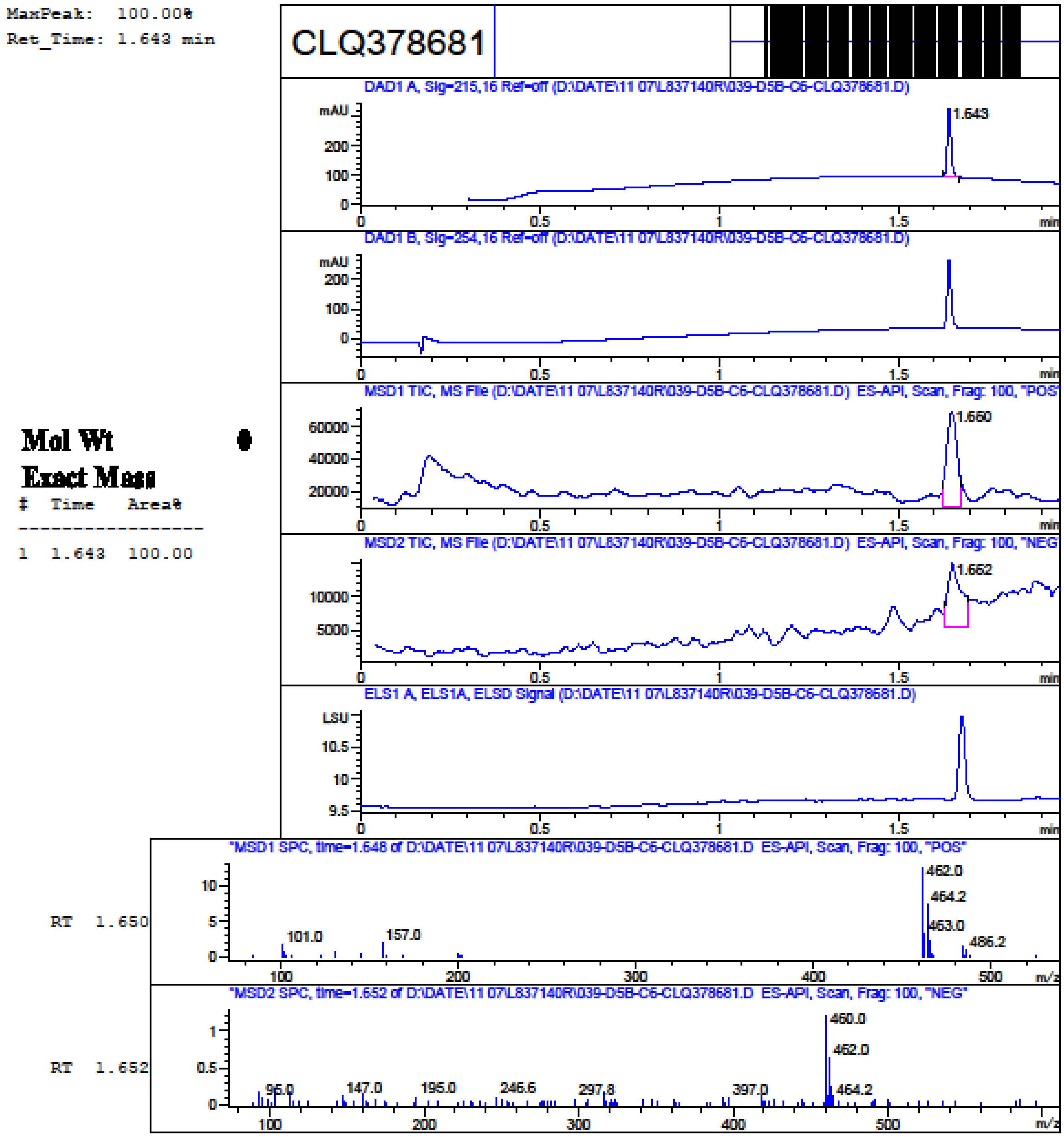

Fig. S8. LC/MS spectrum of compound **2**.

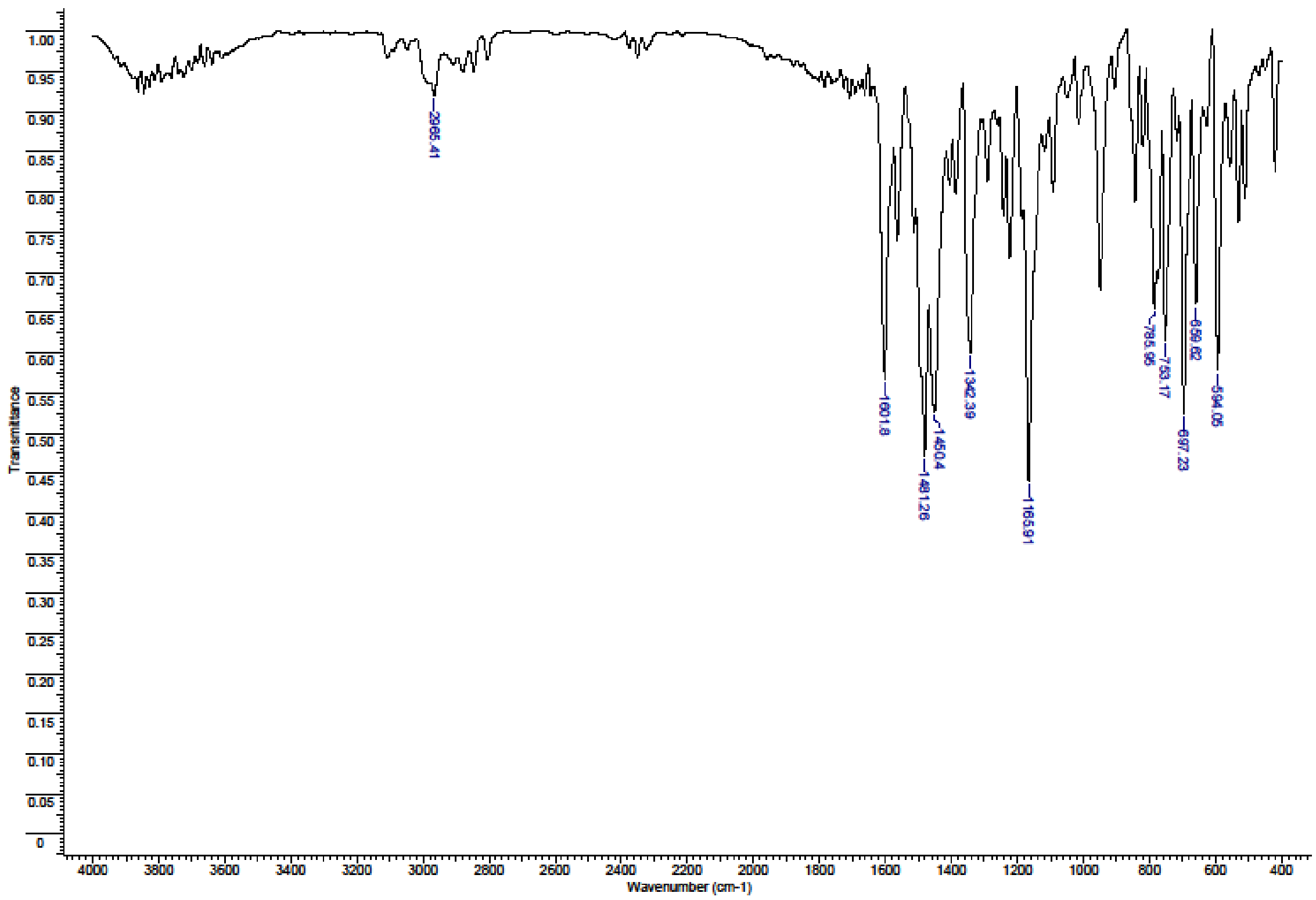

Fig. S9. IR spectrum of compound **3**.

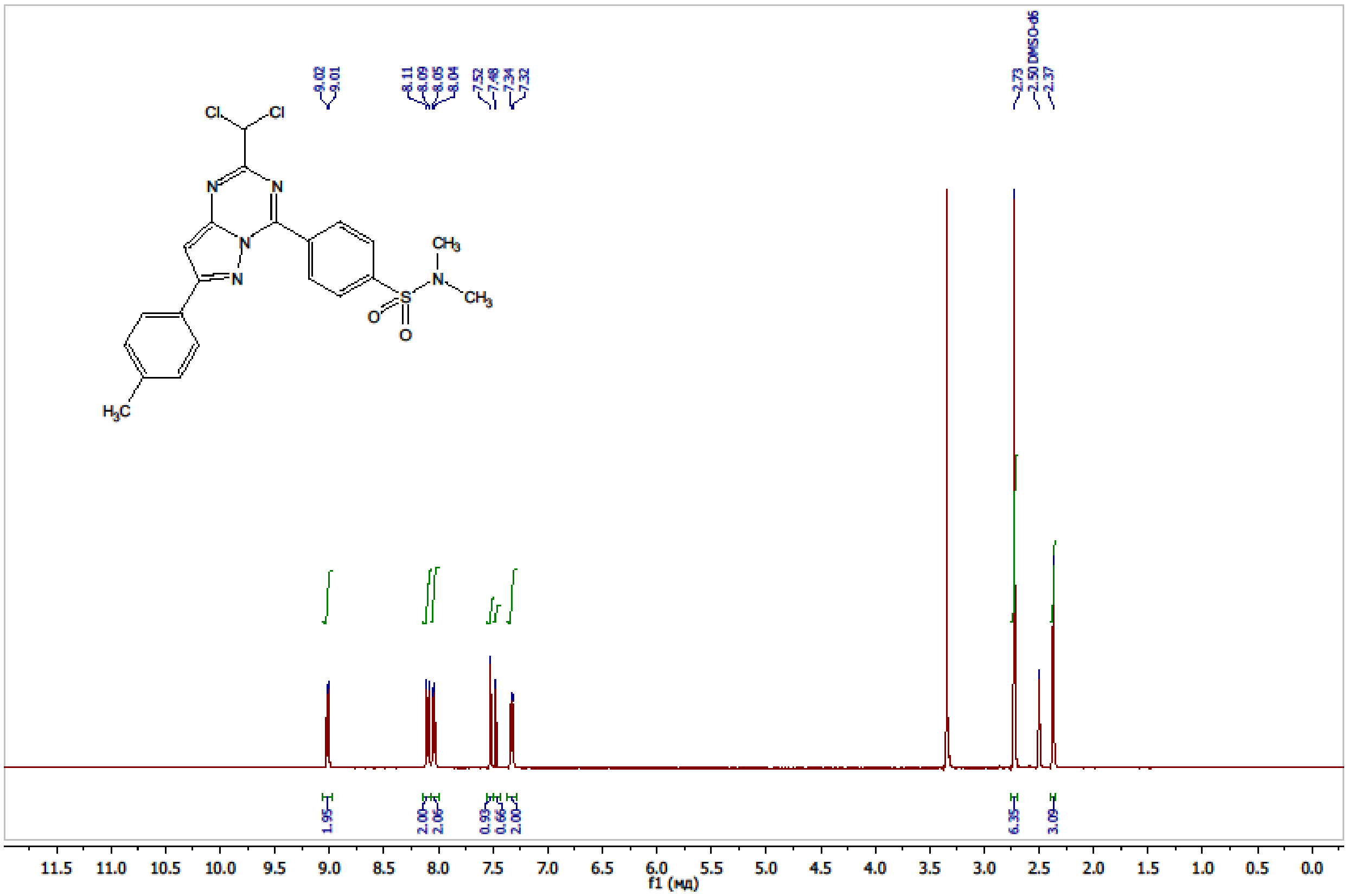

Fig. S10. ^{1}H NMR spectrum of compound **3**.

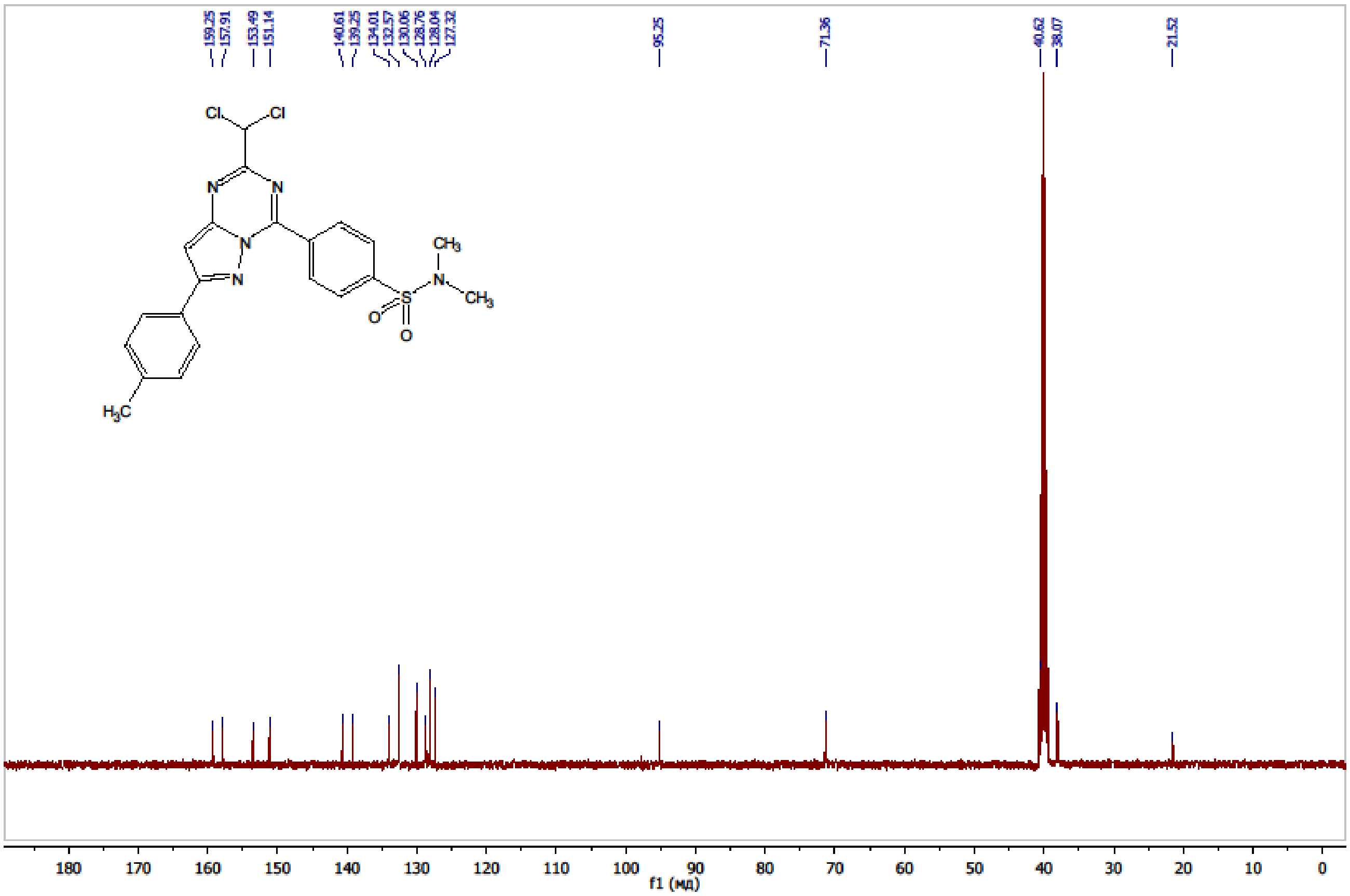

Fig. S11. ^{13}C NMR spectrum of compound **3**.

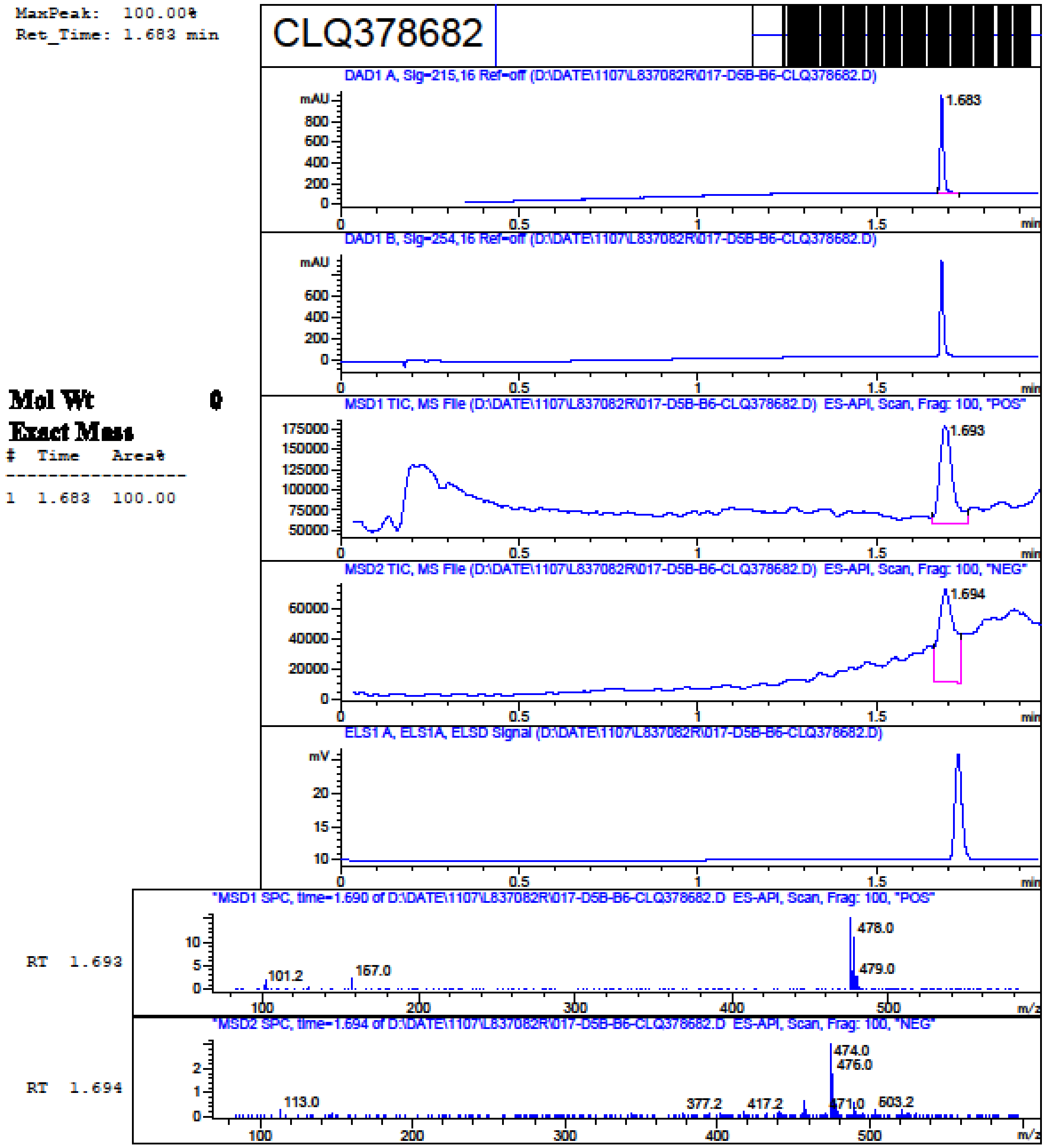

Fig. S12. LC/MS spectrum of compound **3**.

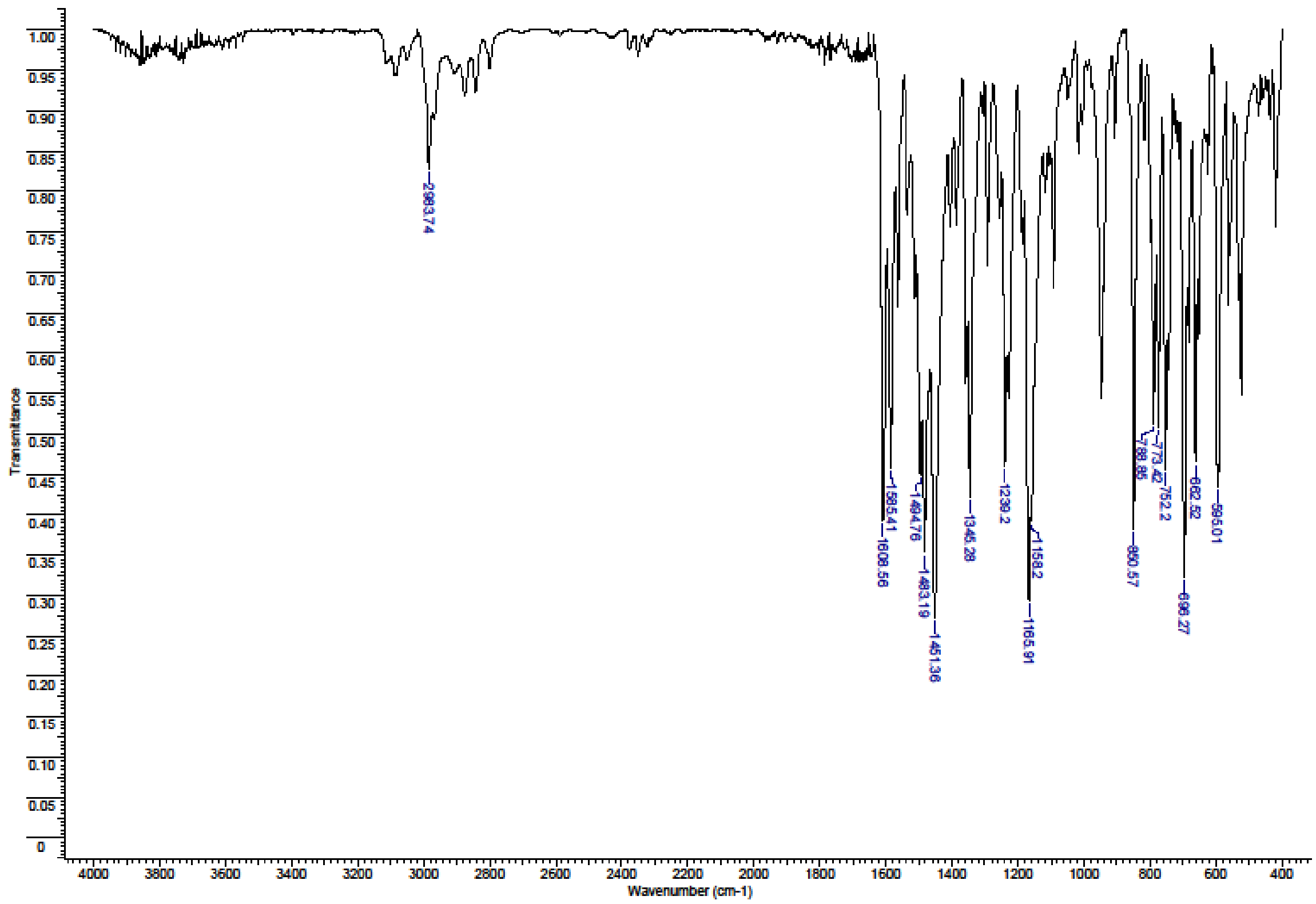

Fig. S13. IR spectrum of compound **4**.

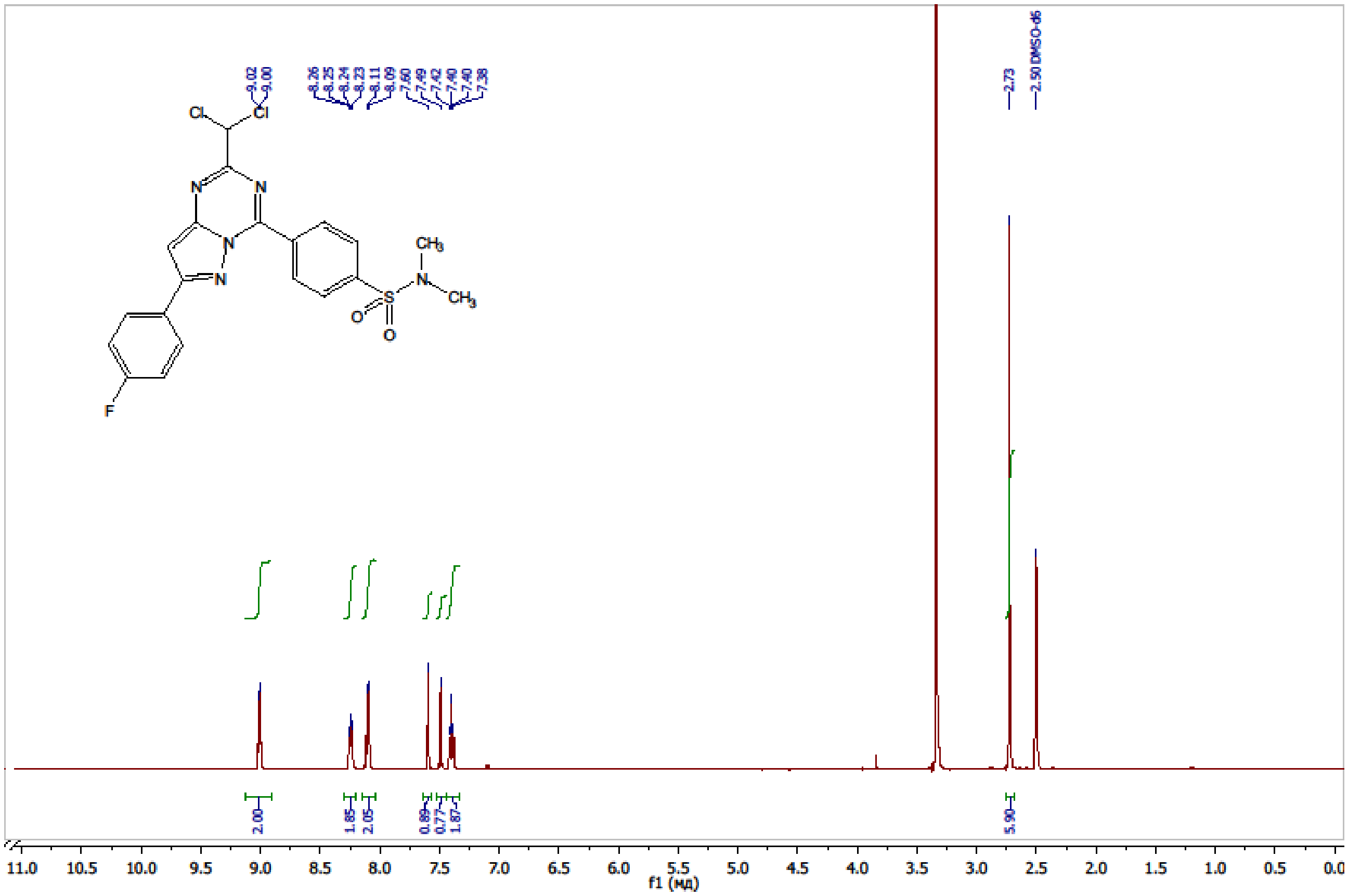

Fig. S14. ^{1}H NMR spectrum of compound **4**.

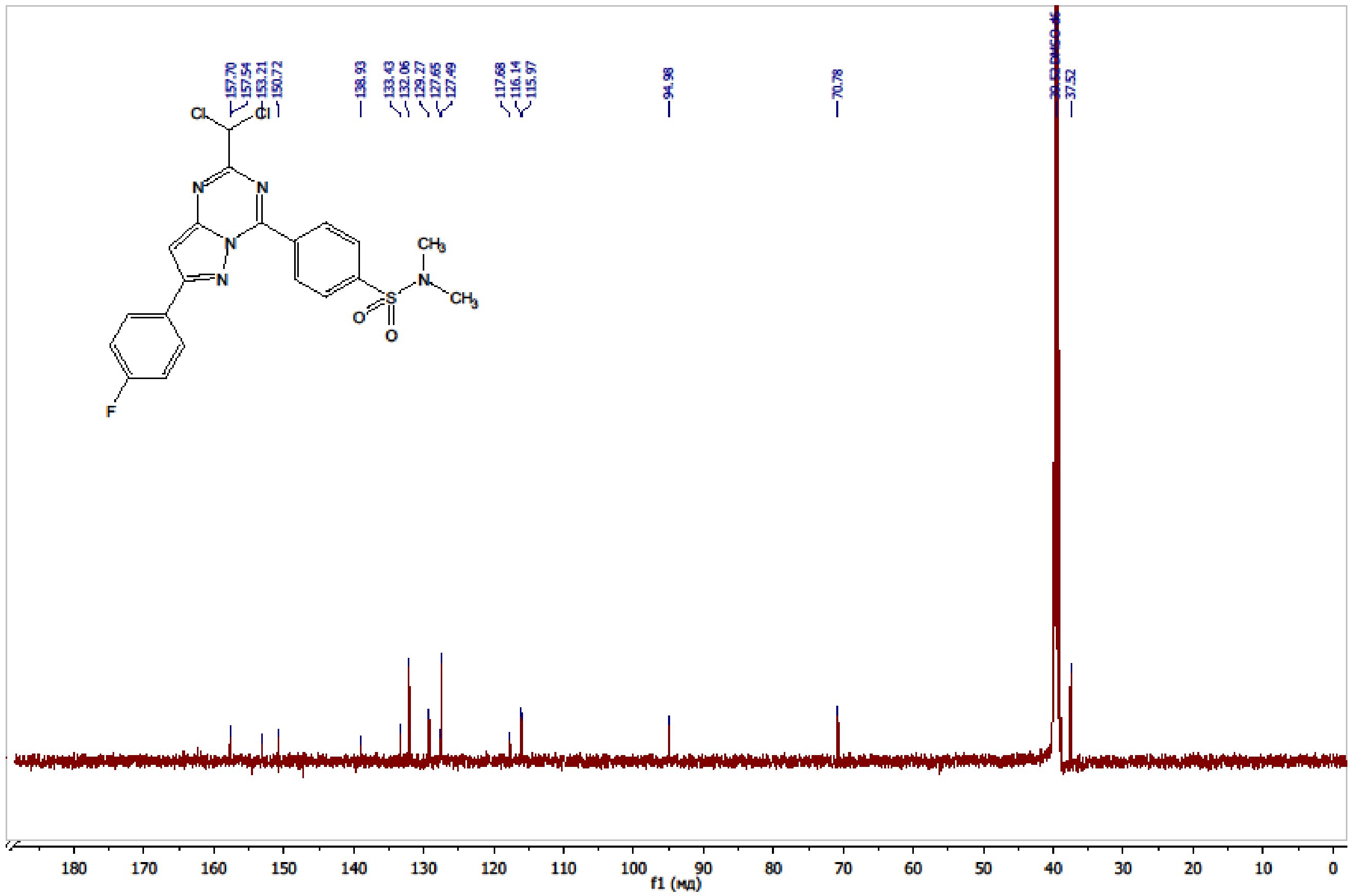

Fig. S15. ^{13}C NMR spectrum of compound **4**.

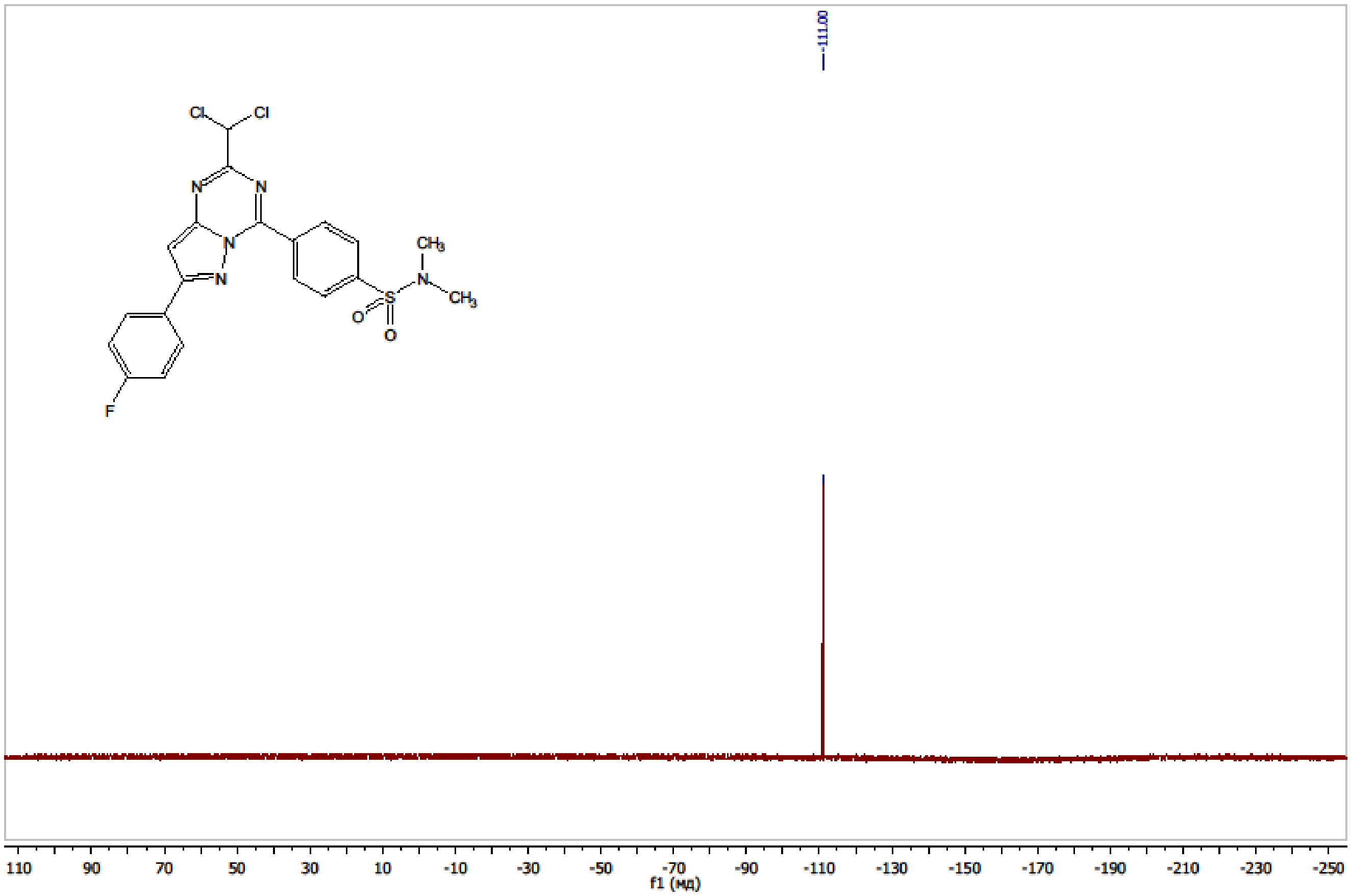

Fig. S16. ^{19}F NMR spectrum of compound **4**.

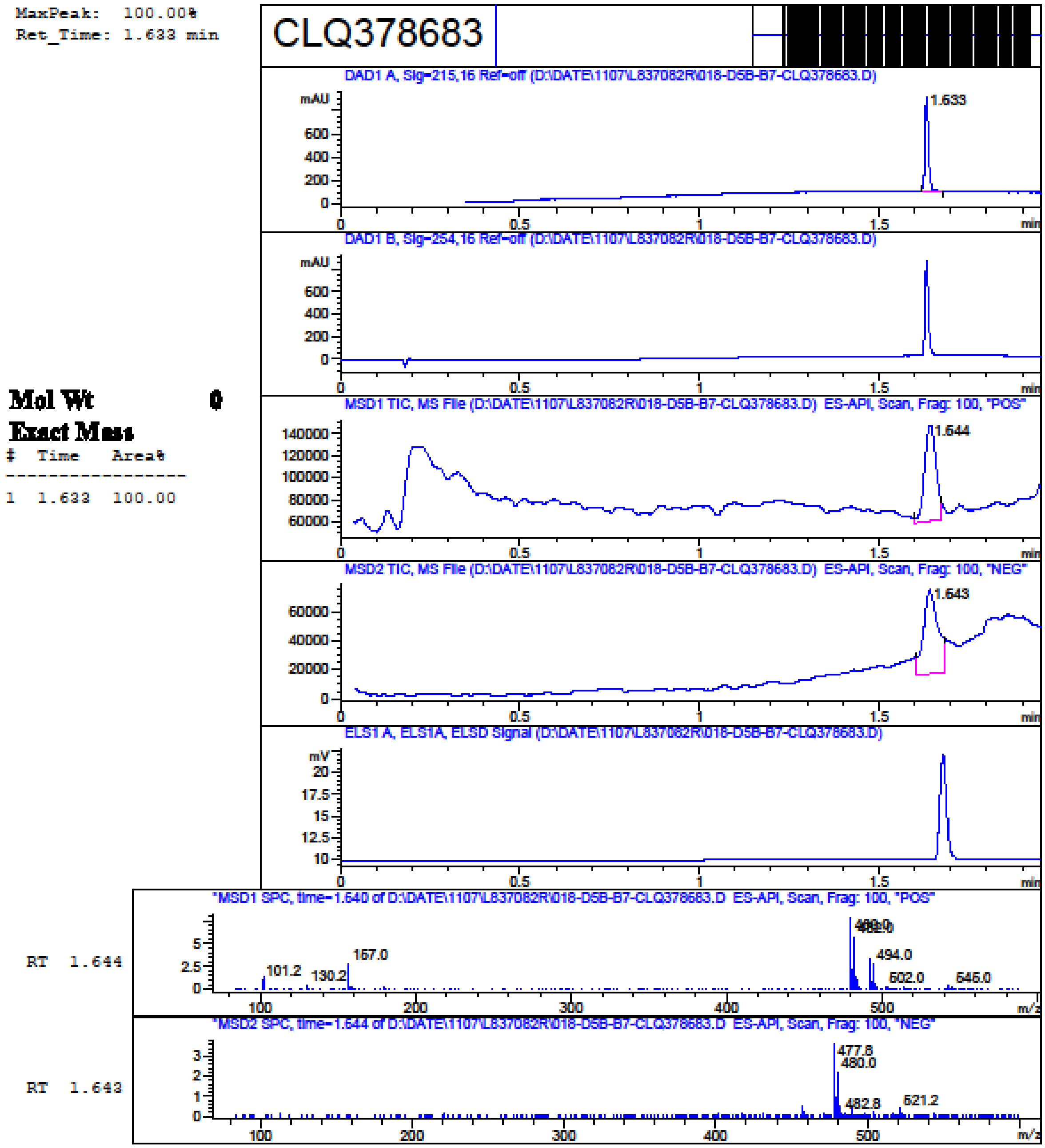

Fig. S17. LC/MS spectrum of compound **4**.

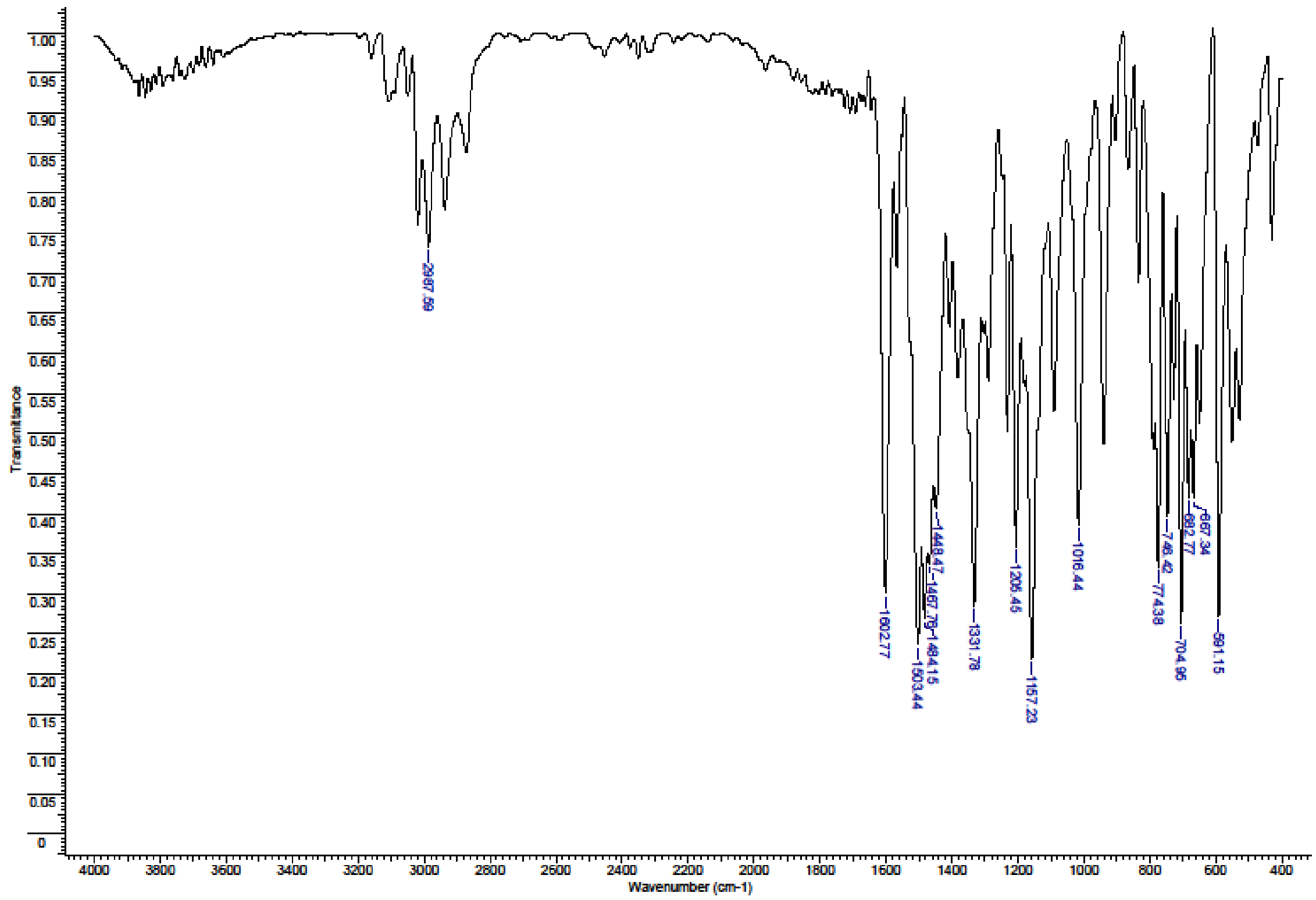

Fig. S18. IR spectrum of compound **5**.

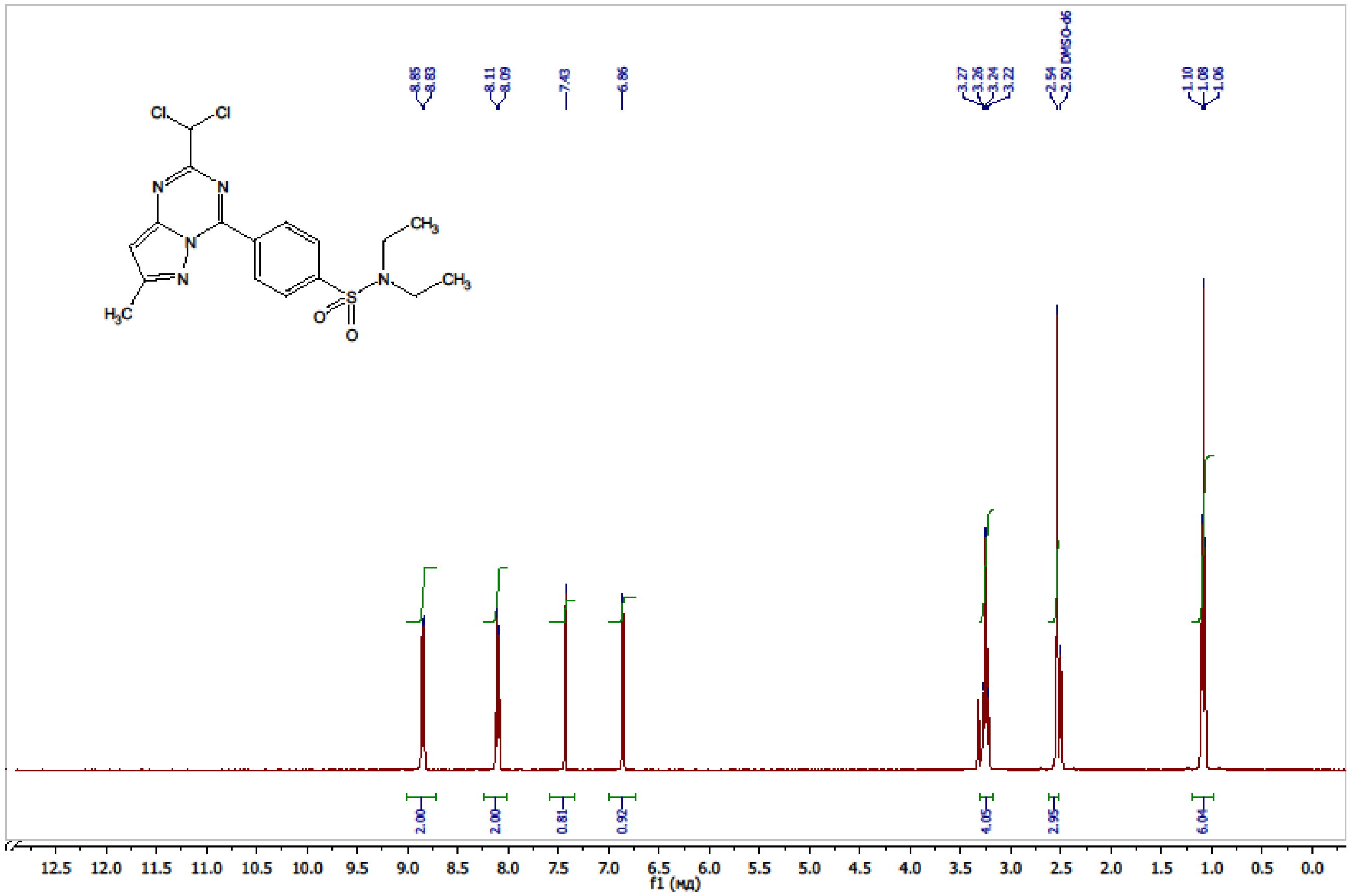

Fig. S19. ^{1}H NMR spectrum of compound **5**.

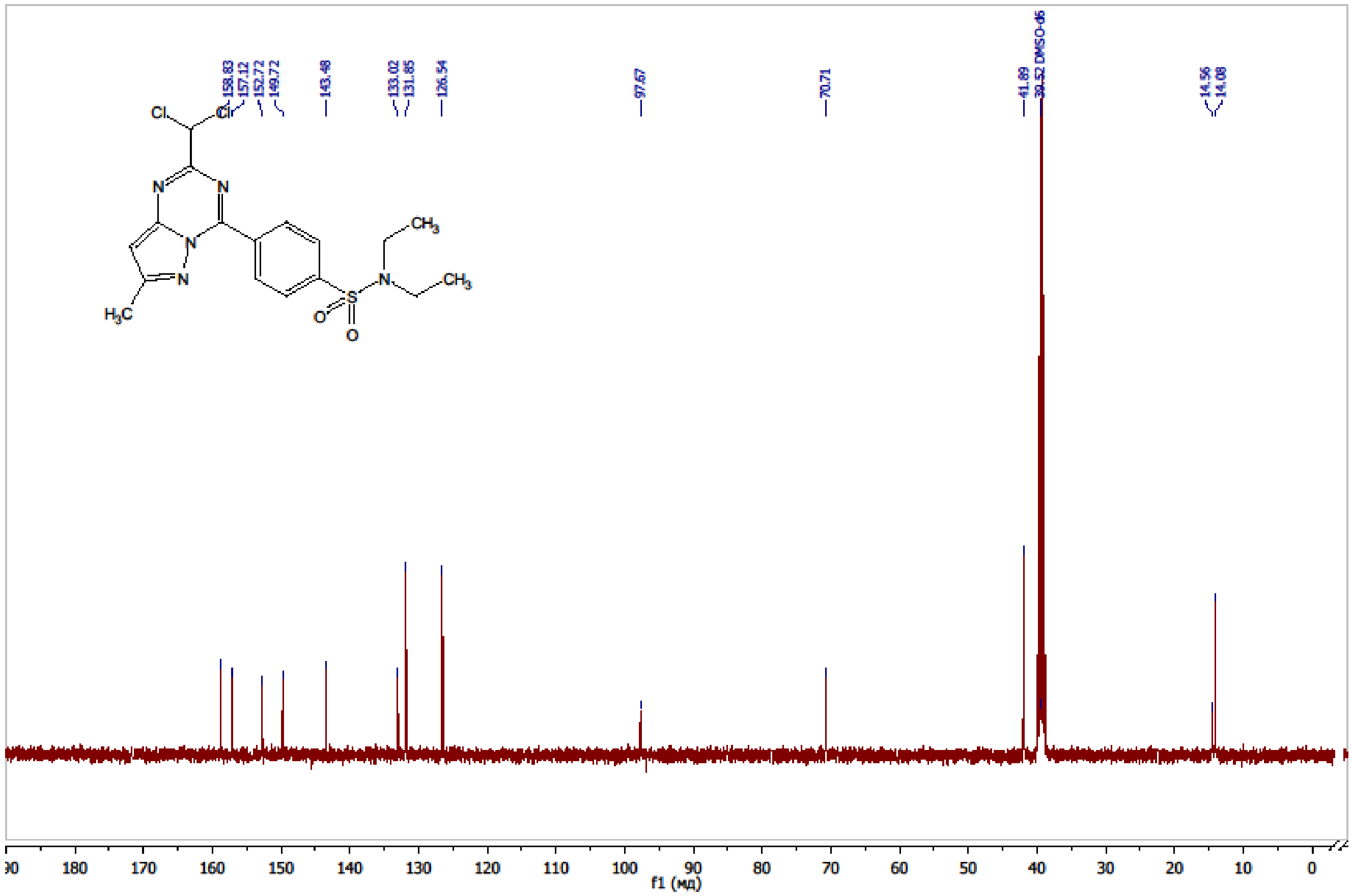

Fig. S20. ^{13}C NMR spectrum of compound **5**.

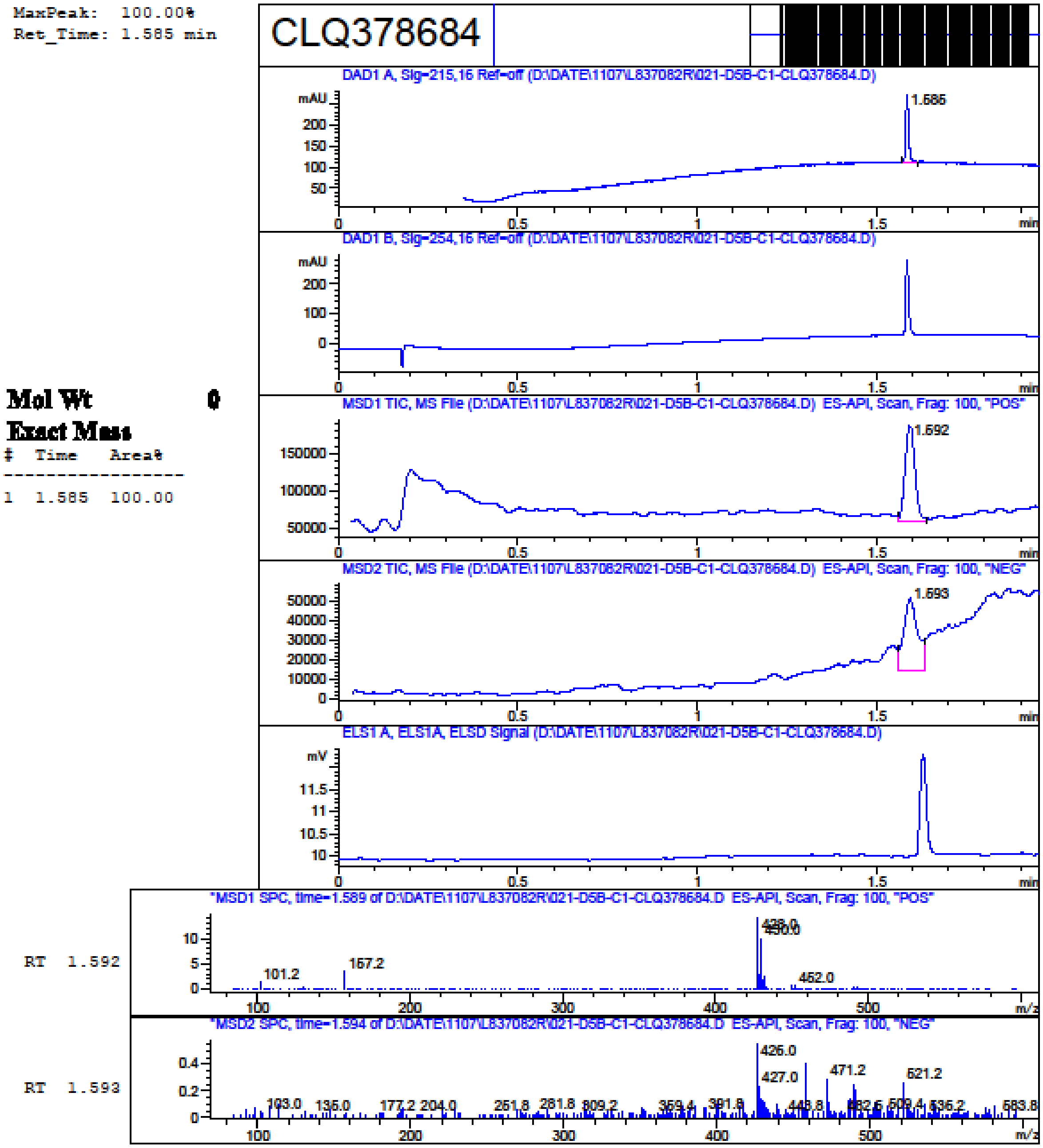

Fig. S21. LC/MS spectrum of compound **5**.

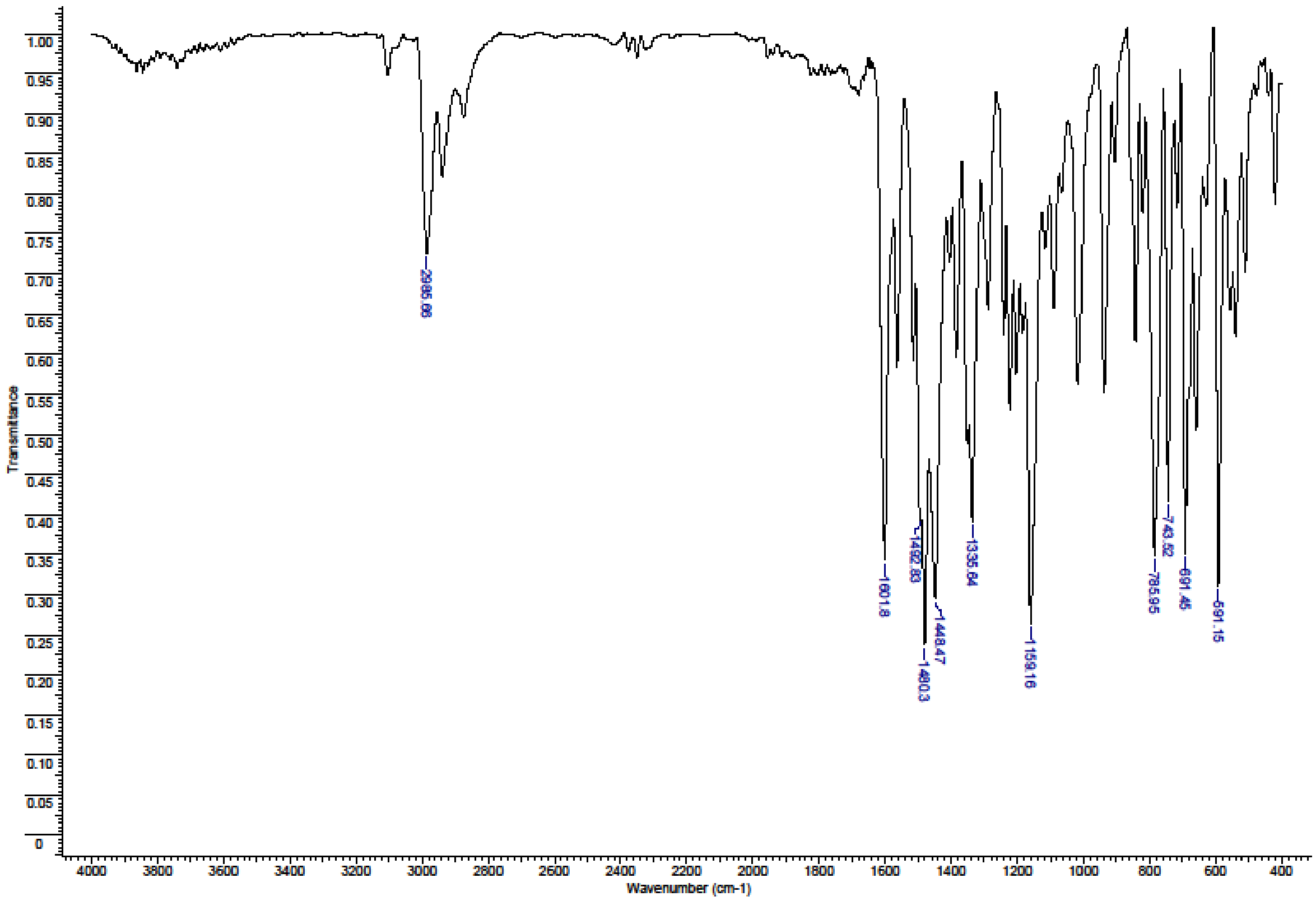

Fig. S22. IR spectrum of compound **6**.

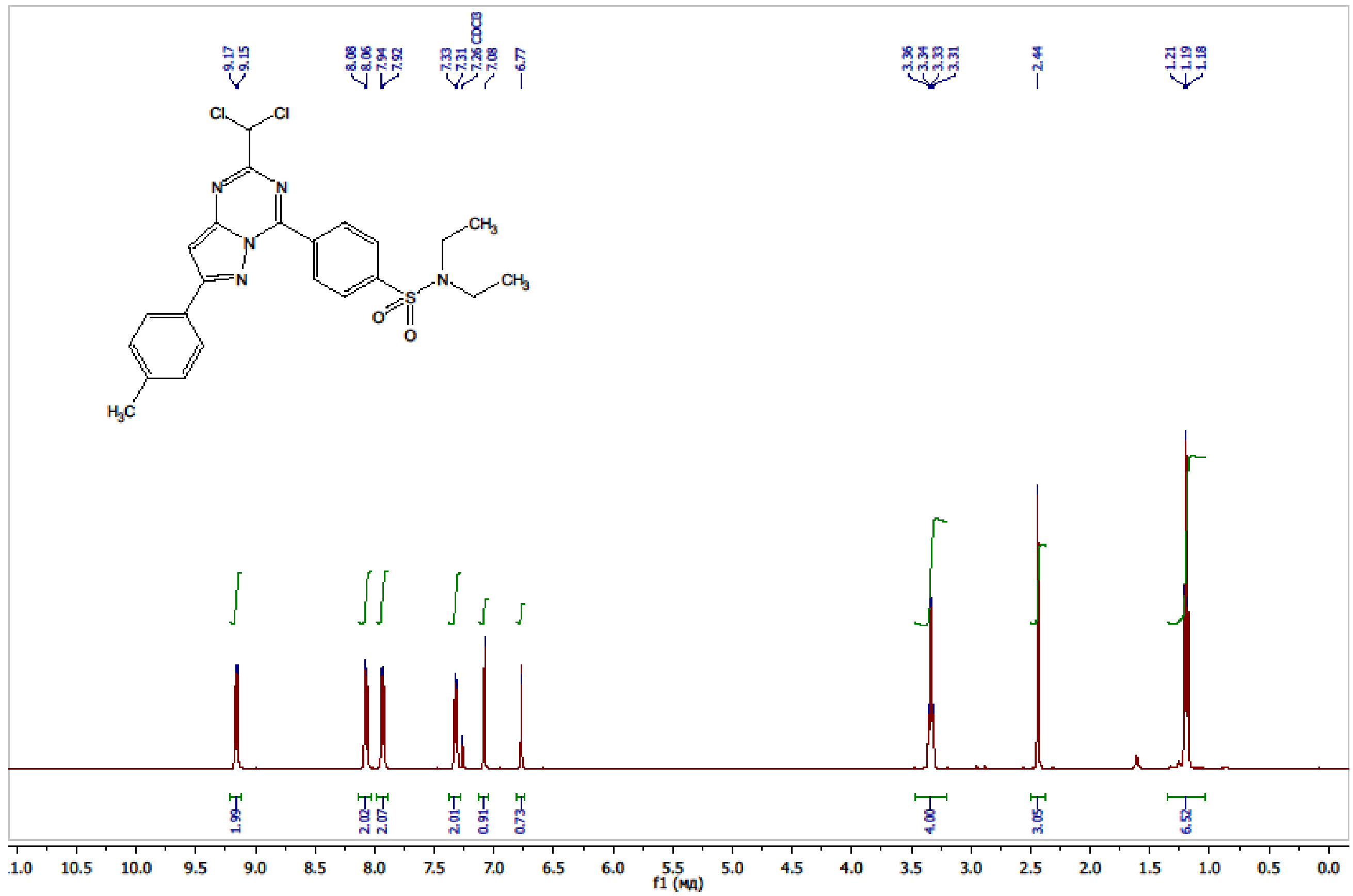

Fig. S23. ^{1}H NMR spectrum of compound **6**.

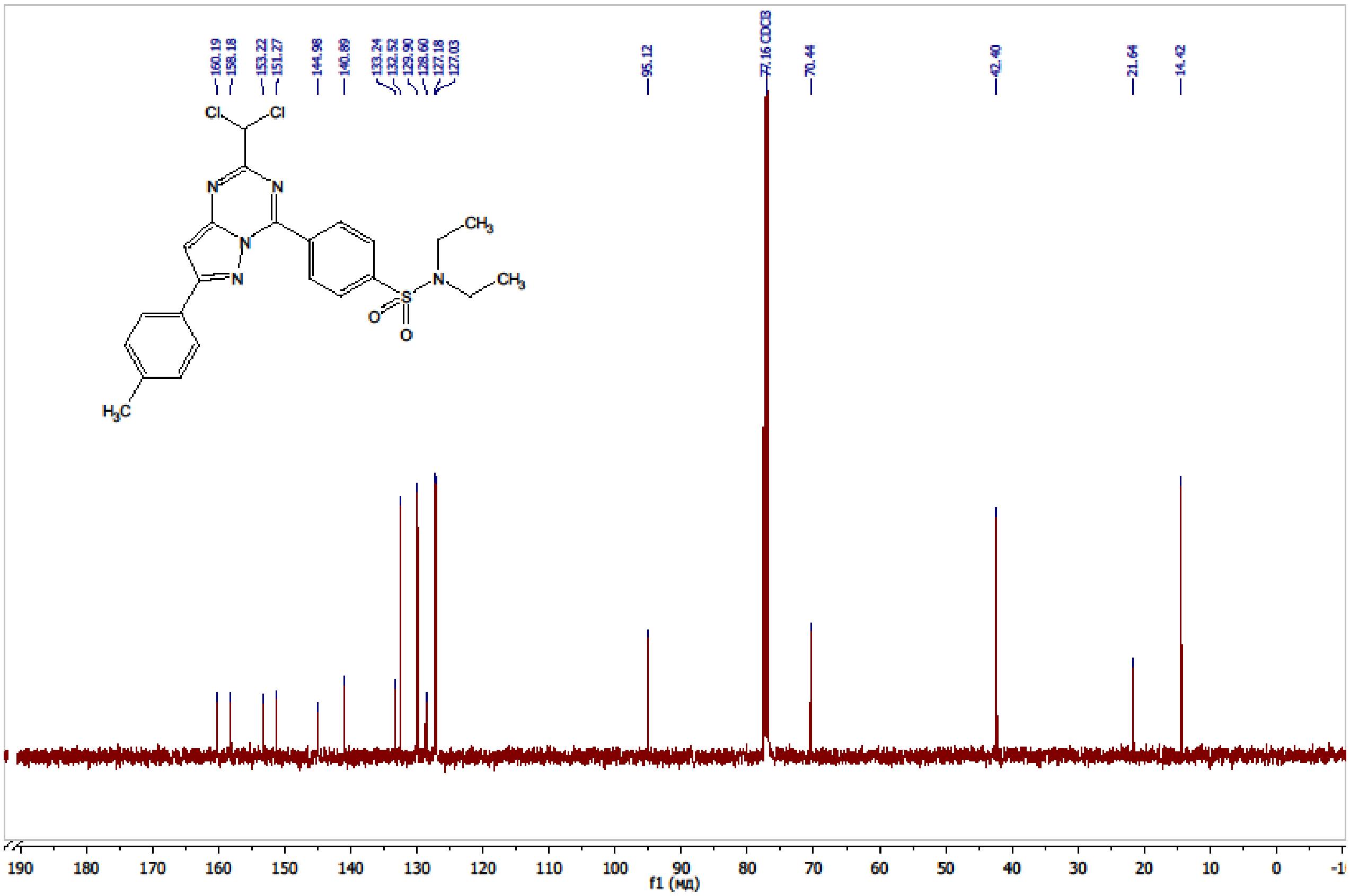

Fig. S24. ^{13}C NMR spectrum of compound **6**.

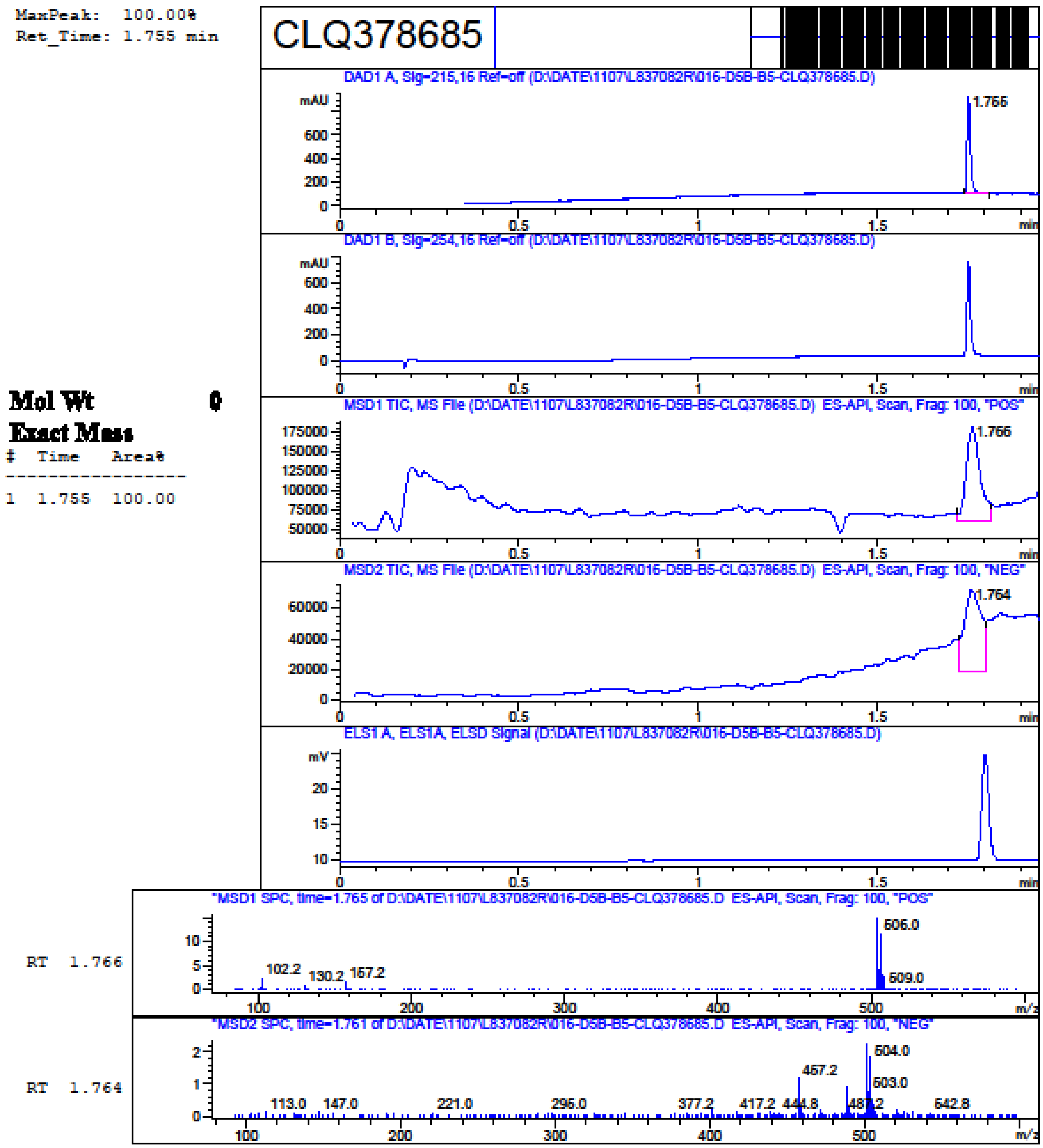

Fig. S25. LC/MS spectrum of compound **6**.

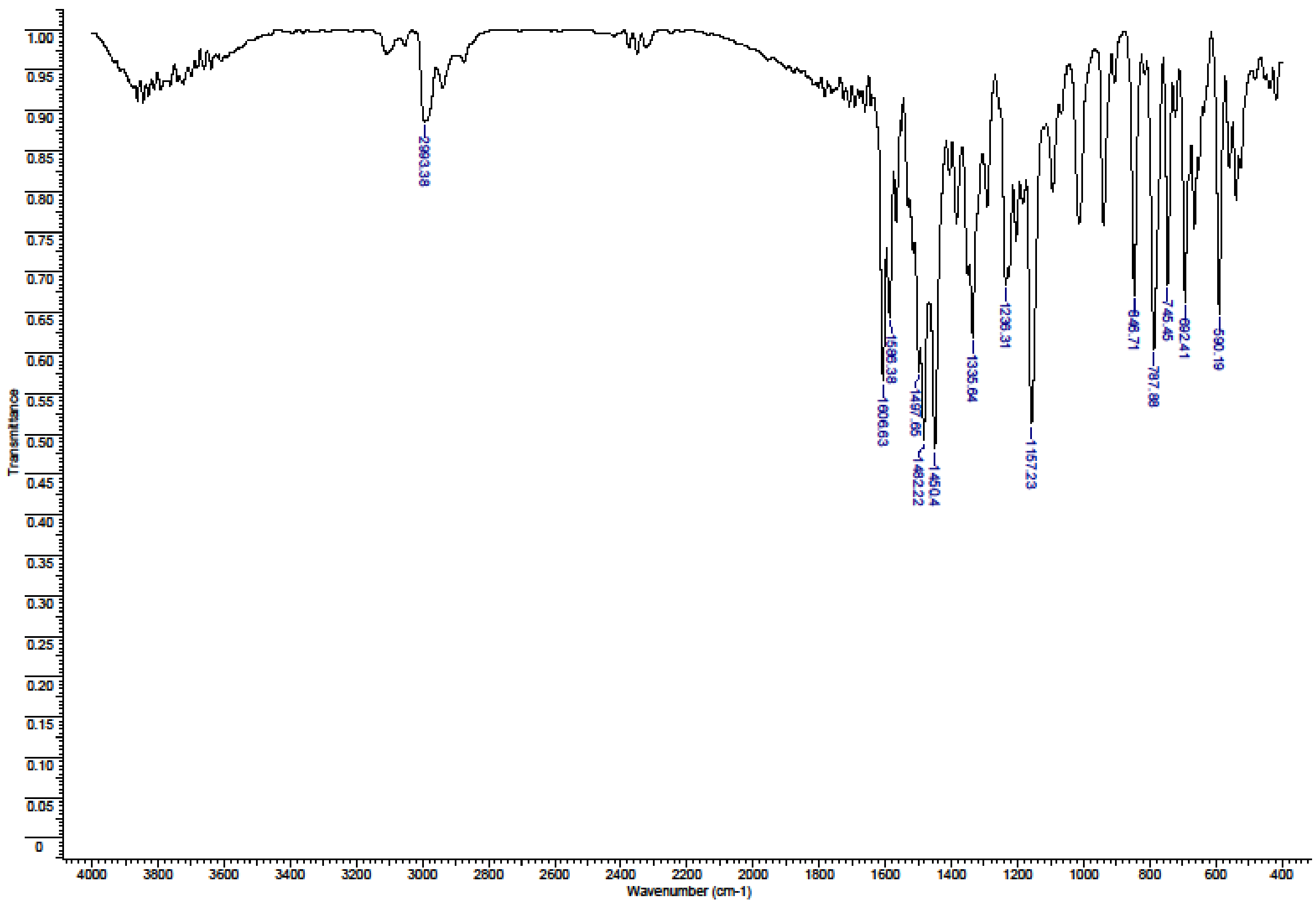

Fig. S26. IR spectrum of compound 7.

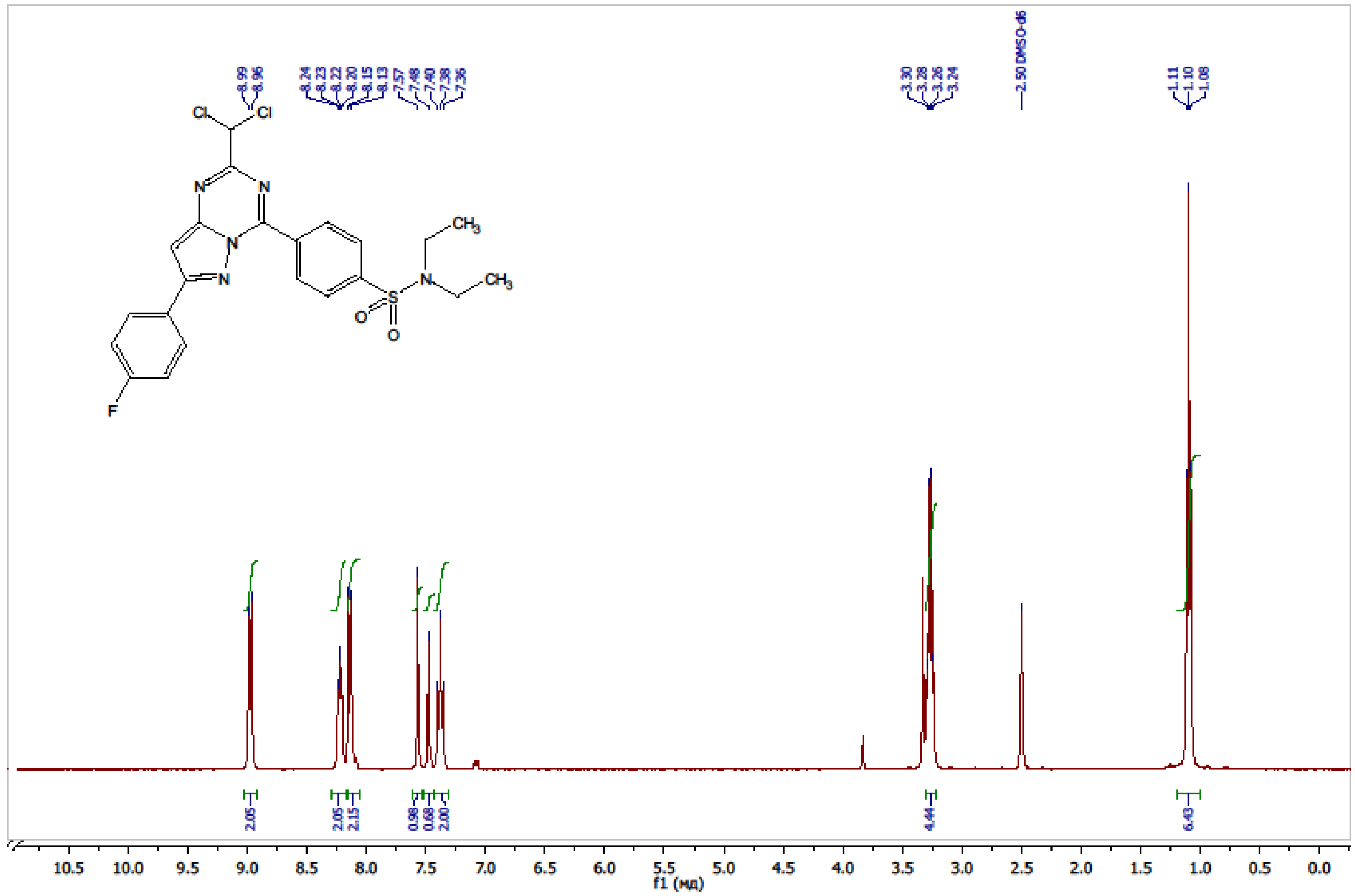

Fig. S27. ^{1}H NMR spectrum of compound 7.

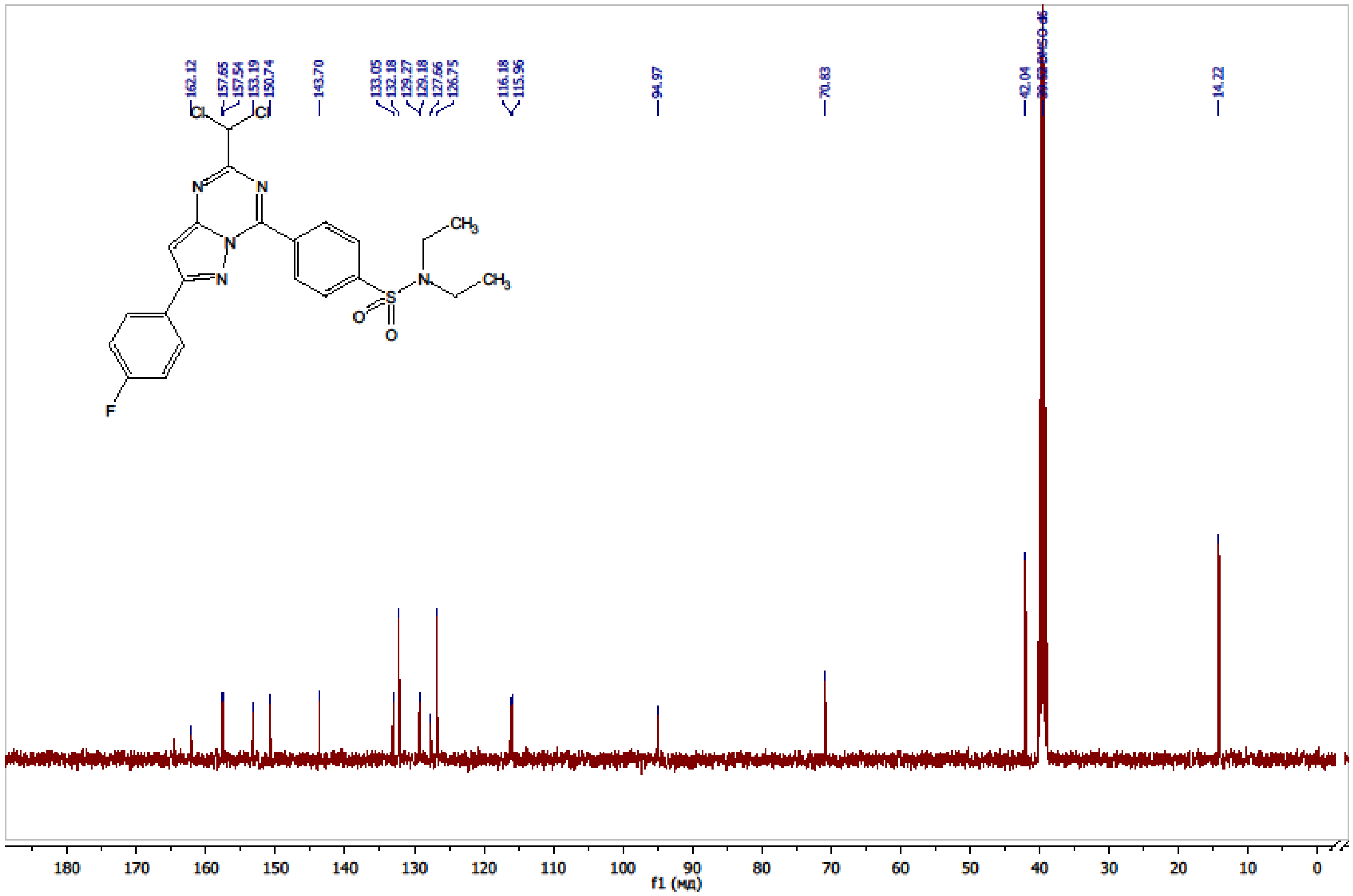

Fig. S28. ^{13}C NMR spectrum of compound 7.

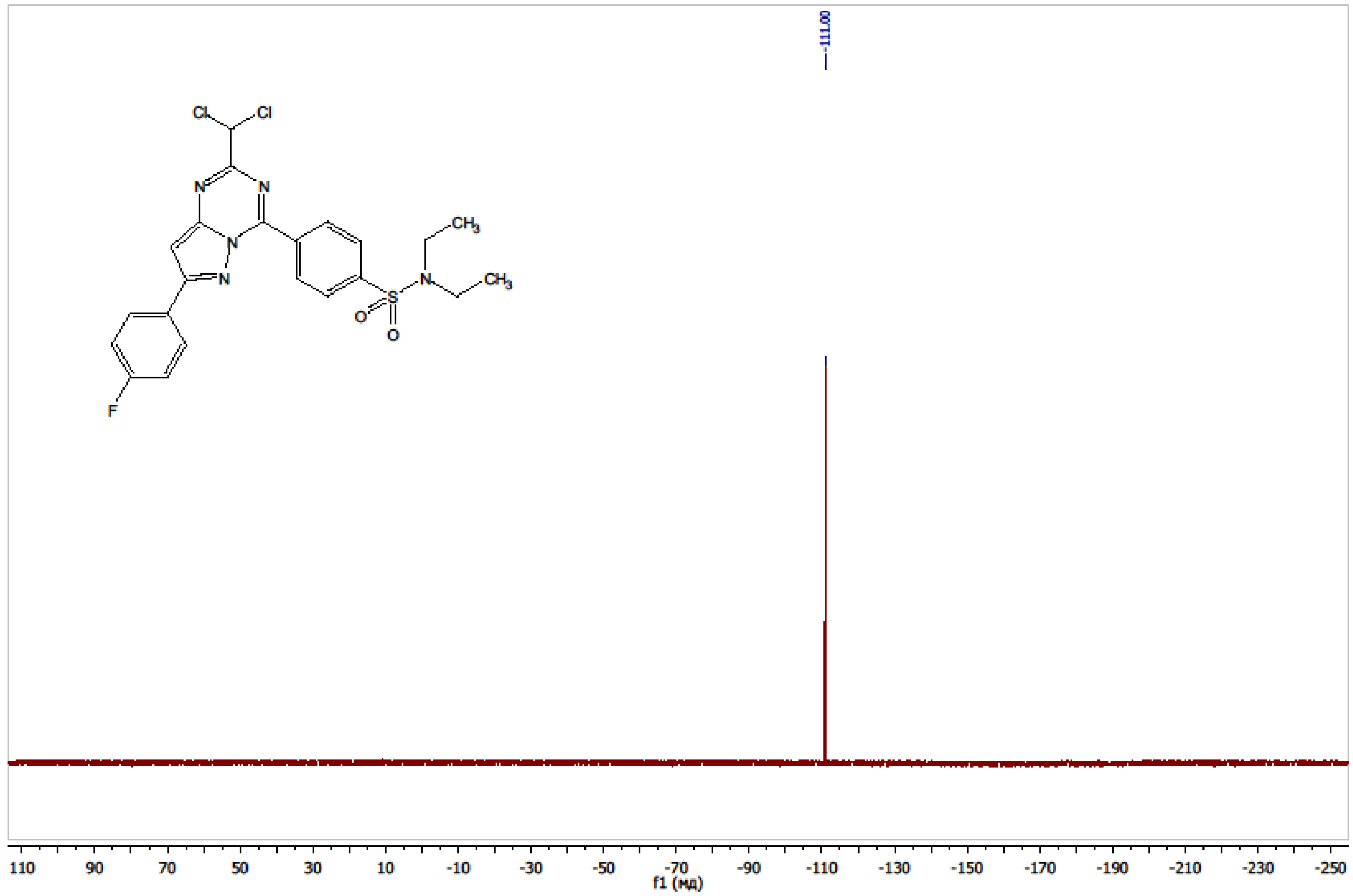

Fig. S29. ^{19}F NMR spectrum of compound 7.

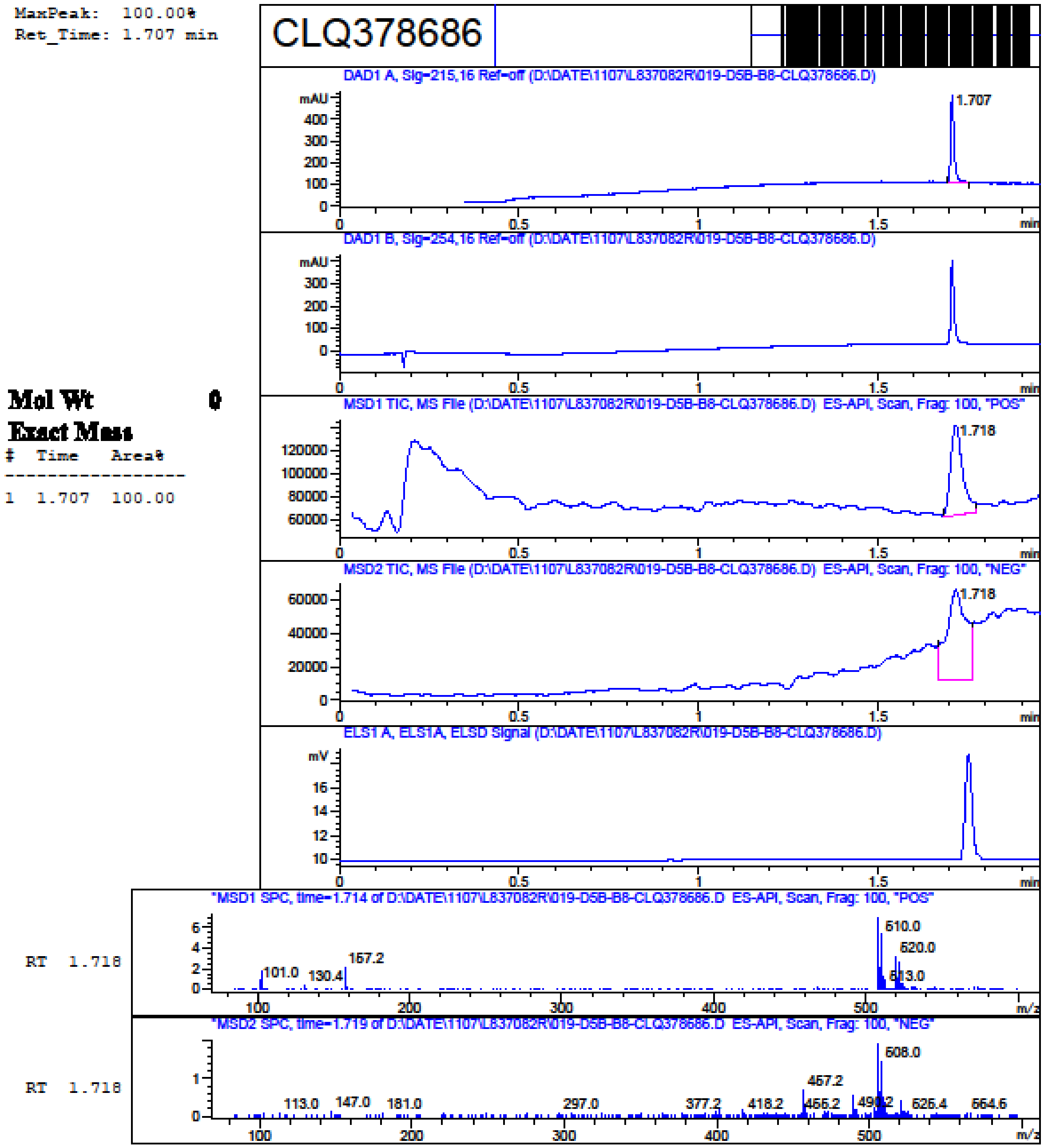

Fig. S30. LC/MS spectrum of compound 7.

Table S3. Growth inhibition of NCI-60 cancer cell lines of synthesized compounds (*one-dose assay*), GI, %

Panel/Cell Line	Compounds						
	1	2	3	4	5	6	7
**Leukemia**							
CCRF-CEM	40.80	77.25	69.31	21.73	48.94	79.21	73.92
HL-60(TB)	0.87	71.09	74.85	6.96	-9.17	70.82	4.64
K-562	14.08	52.21	41.04	8.44	8.36	78.51	34.62
MOLT-4	28.45	63.39	75.57	3.19	36.08	89.00	56.61
RPMI-8226	55.35	86.81	86.88	7.75	-7.21	101.03	6.42
SR	11.77	36.25	68.70	8.24	9.52	84.74	30.79
**NSCLC**							
A549/ATCC	37.01	82.62	88.36	7.50	0.55	95.00	17.78
EKVX	48.75	79.98	102.62	22.65	25.80	106.27	32.01
HOP-62	32.76	70.68	77.86	9.75	5.60	107.89	10.25
HOP-92	49.96	79.03	128.21	14.61	-5.03	105.54	-3.41
NCI-H226	71.77	95.99	87.70	45.35	69.67	109.78	77.28
NCI-H23	44.40	85.01	90.86	-3.27	14.59	98.78	21.71
NCI-H322M	69.33	99.00	107.02	30.99	57.07	112.63	41.41
NCI-H460	27.45	89.05	93.54	1.94	8.65	100.74	10.22
NCI-H522	76.94	89.61	89.68	-16.54	56.05	92.95	79.33
**Colon Cancer**							
COLO 205	25.16	69.50	98.05	2.25	-18.66	117.91	8.90
HCC-2998	47.55	100.41	91.71	8.78	23.75	104.50	7.38
HCT-116	18.18	72.29	62.87	-0.74	10.05	59.08	28.61
HCT-15	5.58	47.74	74.91	-35.68	1.86	91.20	23.34
HT29	17.98	45.64	66.54	-5.58	5.07	91.59	4.77
KM12	39.05	74.04	86.47	7.53	8.52	97.23	9.84
SW-620	18.22	49.60	83.11	4.42	6.12	81.47	23.48
**CNS Cancer**							
SF-268	67.62	88.22	98.70	19.37	56.12	97.58	48.52
SF-295	66.25	86.72	86.19	21.77	48.04	93.95	32.65
SF-539	69.43	77.78	80.25	31.74	50.65	94.67	18.43
SNB-19	58.41	85.21	89.38	23.50	46.19	96.75	40.20

SNB-75	55.49	84.68	107.81	48.31	46.66	105.56	56.53
U251	39.19	66.20	83.78	2.76	8.28	95.38	17.67
**Melanoma**							
LOX IMVI	17.69	44.47	45.73	-6.07	35.63	68.70	32.03
MALME-3M	17.34	15.33	100.42	-11.52	-7.09	97.78	57.59
M14	37.15	74.00	93.11	1.95	4.27	101.42	47.10
MDA-MB-435	47.00	81.58	88.57	6.53	14.85	98.02	25.62
SK-MEL-2	93.02	112.02	112.73	9.25	89.36	117.47	82.30
SK-MEL-28	59.32	-4.08	99.51	27.10	23.28	106.46	27.93
SK-MEL-5	80.28	92.04	91.49	55.14	78.28	101.60	83.25
UACC-257	86.96	104.66	99.97	52.38	59.29	112.40	67.67
UACC-62	68.53	11.71	88.85	25.97	49.85	93.47	54.24
**Ovarian Cancer**							
IGROV1	23.10	66.02	94.98	0.61	12.01	96.56	56.86
OVCAR-3	78.40	93.48	107.51	8.15	21.07	112.07	16.80
OVCAR-4	57.15	90.48	94.30	21.06	34.37	106.09	45.21
OVCAR-5	67.28	89.96	105.69	18.30	41.55	102.94	51.61
OVCAR-8	33.92	60.71	65.49	2.70	12.32	86.74	27.57
NCI/ADR-RES	26.31	69.51	78.81	3.66	14.03	92.91	23.52
SK-OV-3	50.48	92.18	101.04	0.22	18.42	108.63	42.67
**Renal Cancer**							
786-0	94.45	90.62	97.91	72.58	92.53	97.46	76.52
A498	90.89	106.07	100.71	49.67	74.81	110.14	56.23
ACHN	25.22	43.38	83.19	5.28	8.70	102.89	0.48
CAKI-1	44.99	73.07	82.19	3.68	29.60	93.47	12.03
RXF 393	59.54	84.36	90.06	-19.15	13.28	106.42	-30.25
SN12C	55.03	81.14	50.75	24.31	58.56	81.57	67.64
TK-10	84.96	100.81	107.26	29.52	79.39	93.63	49.93
UO-31	65.73	88.85	92.12	44.34	46.43	96.70	27.56
**Prostate Cancer**							
PC-3	67.45	87.68	91.66	26.72	31.08	100.75	18.58
DU-145	45.09	79.24	85.29	5.97	15.92	99.12	15.99
**Breast Cancer**							
MCF7	62.05	72.04	74.17	14.97	23.46	81.99	19.35

MDA-MB-231/ATCC	12.55	48.80	95.09	1.26	-0.48	87.63	1.34
HS 578T	72.75	108.57	105.45	20.31	35.64	107.54	36.39
BT-549	38.46	89.48	93.82	38.00	60.87	96.86	28.04
T-47D	69.80	60.72	82.01	-46.19	-33.64	113.41	-31.87
MDA-MB-468	-20.17	72.04	74.17	14.97	23.46	81.99	19.35
